# SWADESH: a multimodal multi-disease brain imaging and neuropsychological database and data analytics platform

**DOI:** 10.3389/fneur.2023.1258116

**Published:** 2023-10-04

**Authors:** Pravat K. Mandal, Komal Jindal, Saurav Roy, Yashika Arora, Shallu Sharma, Shallu Joon, Anshika Goel, Zoheb Ahasan, Joseph C. Maroon, Kuldeep Singh, Kanika Sandal, Manjari Tripathi, Pooja Sharma, Avantika Samkaria, Shradha Gaur, Sandhya Shandilya

**Affiliations:** ^1^Neuroimaging and Neurospectroscopy (NINS) Laboratory, National Brain Research Centre, Gurgaon, India; ^2^Florey Institute of Neuroscience and Mental Health, Melbourne School of Medicine Campus, Melbourne, VIC, Australia; ^3^Department of Neurosurgery, University of Pittsburgh Medical School, Pittsburgh, PA, United States; ^4^Department of Neurology, All India Institute of Medical Sciences, New Delhi, India; ^5^Medanta Institute of Education and Research, Medanta-The Medicity Hospital, Gurgaon, India

**Keywords:** Alzheimer’s, Parkinson’s, MR spectroscopy, database, glutathione, iron, data analytics, KALPANA

## Abstract

Multimodal neuroimaging data of various brain disorders provides valuable information to understand brain function in health and disease. Various neuroimaging-based databases have been developed that mainly consist of volumetric magnetic resonance imaging (MRI) data. We present the comprehensive web-based neuroimaging platform “SWADESH” for hosting multi-disease, multimodal neuroimaging, and neuropsychological data along with analytical pipelines. This novel initiative includes neurochemical and magnetic susceptibility data for healthy and diseased conditions, acquired using MR spectroscopy (MRS) and quantitative susceptibility mapping (QSM) respectively. The SWADESH architecture also provides a neuroimaging database which includes MRI, MRS, functional MRI (fMRI), diffusion weighted imaging (DWI), QSM, neuropsychological data and associated data analysis pipelines. Our final objective is to provide a master database of major brain disease states (neurodegenerative, neuropsychiatric, neurodevelopmental, and others) and to identify characteristic features and biomarkers associated with such disorders.

## Introduction

The investigation of brain structural, neurochemical and microenvironmental changes using non-invasive neuroimaging techniques is crucial to understand brain function in health and disease. Neuroimaging databases from all over the world are helping to advance such brain research. For instance, the Alzheimer’s Disease Neuroimaging Initiative (ADNI) was developed for sharing volumetric magnetic resonance imaging (MRI) data (1). Similarly, SchizConnect is an open database that hosts structural, diffusion profile, and functional MRI data for Schizophrenics (2). The Parkinson Progression Marker Initiative (PPMI) (3) and Quebec Parkinson Network (QPN) (4) are two independent multimodal neuroimaging databases involving control subjects and patients with Parkinson’s disease (PD). There are also databases for autism (5), bipolar (6), epilepsy (7), and other neurological disorders. Most of these databases primarily contain structural information using MRI modality. Other MR techniques such as functional MRI (fMRI), quantitative susceptibility mapping (QSM), magnetic resonance spectroscopy (MRS) and diffusion weighted imaging (DWI) offer additional functional, susceptibility-related, neurochemical, and microstructural information, respectively. This diverse set of information is crucial for conducting comprehensive analyses of various brain disorders.

MRI based imaging provides volumetric changes and tissue texture alterations. DWI provides the microstructural changes from the movement of water molecule primarily in the white matter (8–10). fMRI provides the functional changes in specific anatomical regions exclusively due to blood oxygen level dependent (BOLD) signal changes (11–13). These imaging-based features represent structural changes and/or functional impairments, but usually cannot discern causal factors. However, combined features from MRS (14–19) and brain susceptibility information (generated using QSM) likely provide an understanding of possible causal molecular processes (20).

It is important to note that no existing database currently encompasses both neurochemical and magnetic susceptibility data that are closely linked to the etiology of brain disorders. The absence and inadequacy of neurochemical and magnetic susceptibility data represent critical limitations within the current databases, as this data plays a significant role in determining the underlying pathology. Consequently, there exists an urgent need to bridge this gap by developing a comprehensive database that incorporates diverse data samples, testing modalities and brain disorders all under one unified platform.

**Figure 1:**
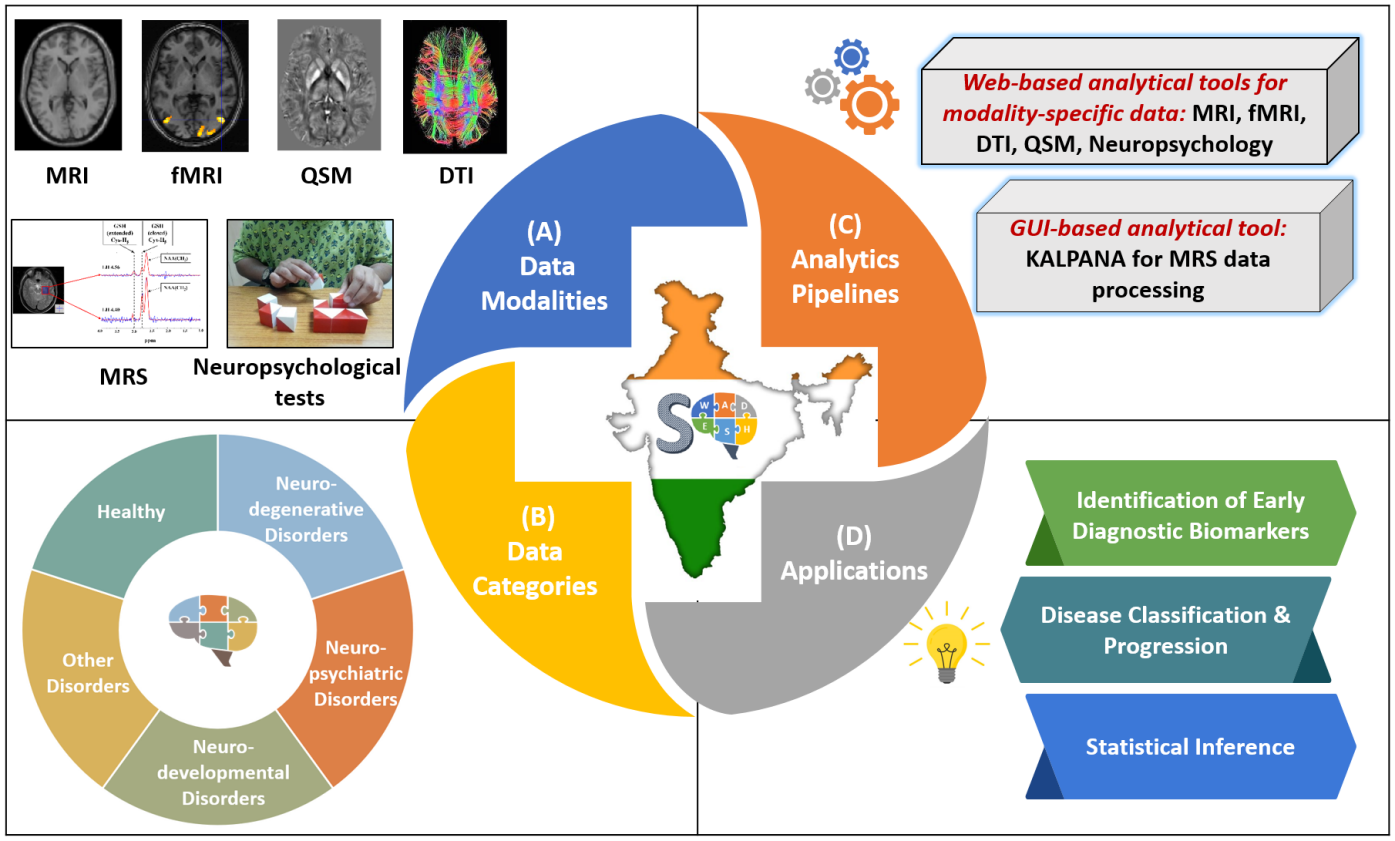
Overview of the SWADESH platform. **(A)** Data modalities: MRI, MRS, fMRI, DTI, QSM, and neuropsychological; **(B)** Data Categories: neurodegenerative, neuropsychiatric, neurodevelopmental, other disorders, and healthy subjects, **(C)** Analytics pipelines: Web-based analytics pipelines for MRI, fMRI, DTI, QSM, and neuropsychological data and KALPANA package for MRS processing. **(D)** Applications in terms of scope of framework.

To address these important points, we have developed SWADESH—a comprehensive web-based platform that encompasses multimodal neuroimaging data along with spectroscopic and neuropsychological information and data analytic pipelines (Figure 1) (20, 21). The platform hosts multimodal neuroimaging and behavioral data for mild cognitive impairment (MCI), Alzheimer’s disease (AD) and Parkinson’s disease (PD) and is accompanied by data processing and analytical tools for each modality as shown in Figure 1.

The major features of SWADESH platform are as follows:

- Comprehensive neuroimaging platform: The platform hosts multimodal (MRI, fMRI, QSM, MRS, DWI, and neuropsychological) and multi-disease (AD, MCI, and PD) data along with modality specific data analytic pipelines on a single platform.
- Data storage and multipoint data sharing: The acquired data is categorized into specific data formats based on the neuroimaging modalities, diseases, spatial, and temporal features. Multiple devices can be connected simultaneously to access the SWADESH platform.
- Data anonymization: To make the data anonymous and to protect the subject’s identity, a type of information sanitization was performed, which made subjects’ information completely shielded from the users.
- Convenient and secure web access: This platform allows access to multimodal neuroimaging and neuropsychological data and their analytical pipelines on a single-user friendly web portal. We have uploaded necessary packages, and no additional software installation is required for the analysis of various data in the SWADESH platform.
- Scalability: SWADESH has the feature to increase or decrease its allocated computational resources based on the number of requests at any given time. It can also be scaled very easily to accommodate more datasets and database tables as per the research requirements. SWADESH has a potential for linking world-wide databases on a single platform, enabling researchers to leverage existing data and analytical resources.
- Data curation by a research team: For continual improvement of SWADESH, a dedicated research team ensures availability, reliability, safety, and maintenance.

## Materials and methods

This section provides the details of the two major components: database and data analytical pipelines.

### Database

#### Storage, accessing, and visualization

The SWADESH platform is developed to organize, visualize, process, and analyze multimodal, disease specific neuroimaging and behavioral data. The whole data is organized as per the modality, along with meta information (such as disease type, gender, age, and anatomical region under study). The users can search this heterogeneous database with meta information as keywords and download the specific data as per their requirements. In this platform, the MRI and fMRI data are available in NIfTI, PAR and REC formats. QSM and DTI data are available in DICOM formats. MRS data is available in SPAR and SDAT format. Three-dimensional (3D) image visualization interface^1^ is added in our platform for visualizing DICOM and NIfTI format data arising from various neuroimaging modalities. This orthogonal viewer is configured with various display, menu, and control options and compatible across a range of web browsers.

#### Data anonymization

Anonymization or de-identification is very important to protect the identity of each participant (22). SWADESH platform has accomplished data anonymity feature by protecting the demographic and personal information of each participant.

### Data analytical pipelines and plugins

A data analytical pipeline is a set of processing steps that are applied to raw data for extracting relevant information. In SWADESH, the neuroimaging data analytical pipelines are divided into two categories: *Web-based Data Analytical Pipeline* and *GUI-based Data Analytical Packages* (Figure 1C).

#### Web-based data analytical pipeline

##### MRI data analysis pipeline

The MRI technique provides three-dimensional structural information of the brain non-invasively. In SWADESH, we have developed an automated MRI processing pipeline using Linux shell scripts. This customized MRI pipeline uses various existing toolboxes such as the Automatic Registration Toolbox (ATRA) (23), Robust Brain Extraction (ROBEX) (24), Medical Image Processing, Analysis, and Visualization (MIPAV) (25), FreeSurfer (26), and Advanced Normalization Tools (ANTS) (27).

**Figure 2:**
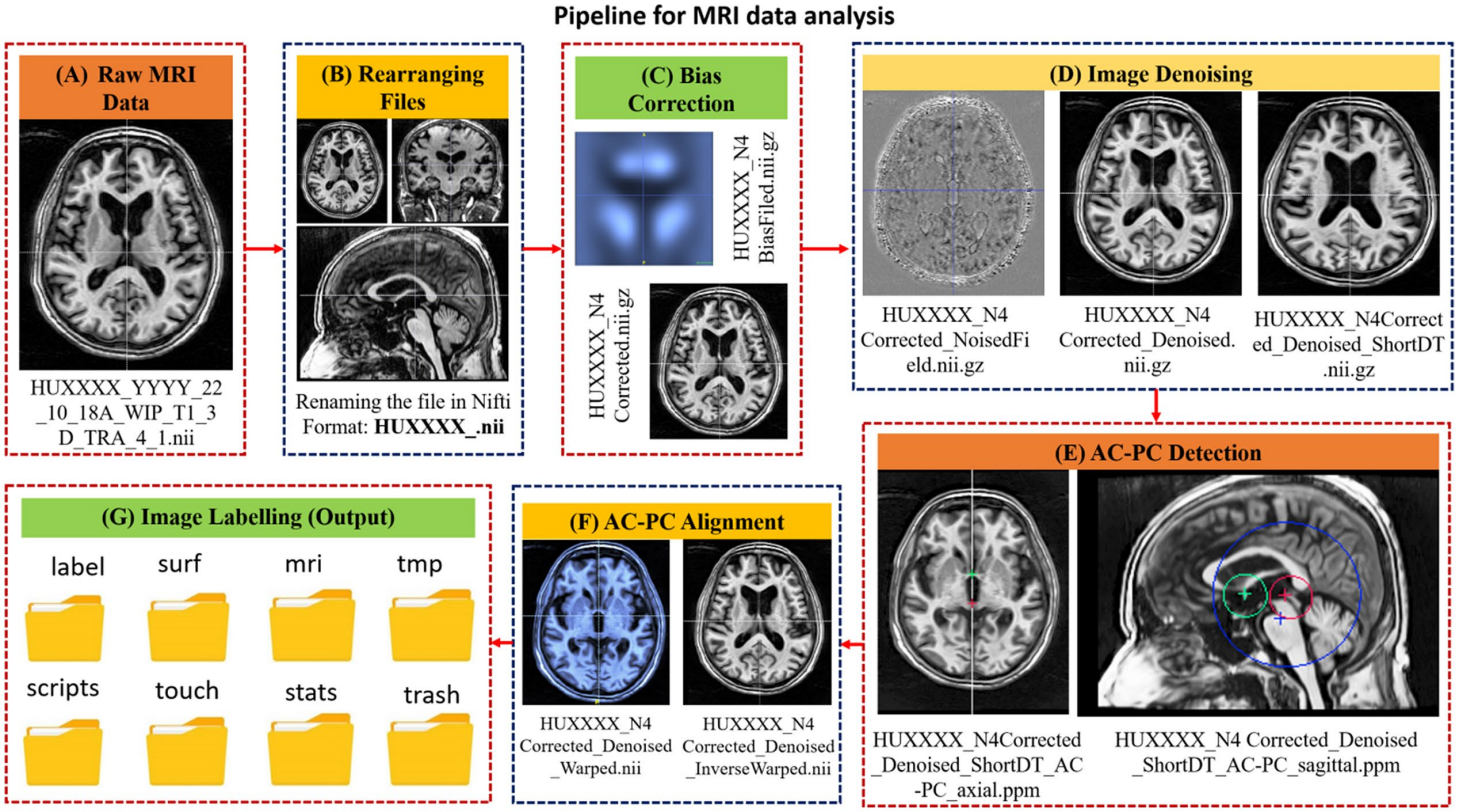
A schematic diagram of the steps involved in the MRI data processing pipeline along with intermediary outcomes. The designed pipeline involves **(A)** landmarks detection on raw image, alignment to landmarks and image labeling. **(B)** Renaming the file in NIfTI format for further processing, **(C)** Application of bias correction **(D)** Image denoising step was applied to detect noise field and to generate denoised image, **(E)** Landmark detection wherein, the AC and PC locations are detected, and a corresponding transformed matrix is generated **(F)** Landmark alignment where, the denoised images are aligned to the AC-PC line. **(G)** Eventually, the labels are generated using recon-all (reconstruction) command of Freesurfer.

In the MRI data analysis pipeline, first the raw data is converted into NIfTI format, followed by bias correction (Figure 2). Low-frequency intensity inhomogeneities are removed from the raw image by implementing the Non-Parametric Non-Uniform Intensity Normalization (N4ITK) algorithm (28) through ANTs. Consequently, a bias-corrected image is obtained. The bias-corrected image is further filtered using Adaptive Non-Local Means (ANLM) filtering through ANTs (29). At this stage, a denoised image with an improved signal-to-noise ratio and the removed noise field is obtained. The denoised images are then processed to locate the anterior commissure (AC) and the posterior commissure (PC) landmarks with the AC-PC line and oriented along the AC-PC line using the MIPAV and ATRA toolboxes (23, 25). Subsequently, the denoised image is firmly registered to the AC-PC detected image generated using the ANTs toolbox. The warped and inverse warped images (to minimize distortions) are obtained with the transform matrix and the inverse warped images. Finally, the anatomical labels are obtained from the AC-PC aligned image using the Freesurfer toolbox in a folder with the name *‘label’* along with seven other folders namely, *MRI scripts*, *stats*, *surf*, *tmp*, *touch* and *trash*. Each file in these output folders has different application and can be utilized for further analysis (30).

##### fMRI data analysis pipeline

Brain activity is assessed in fMRI using BOLD signals (11). Task based and resting state fMRI are commonly used techniques to examine brain functional connectivity (31). Suitable paradigm design, application and post-processing tools for fMRI data have progressed tremendously in recent years (32). For fMRI data processing in SWADESH, MATLAB-based batch scripts were developed which utilized inbuilt functions of the SPM12 toolbox (Wellcome Centre for Human Neuroimaging at University College London) (33). The user can run these scripts directly on the SWADESH platform, without installing the MATLAB and SPM12 toolbox on their system.

**Figure 3:**
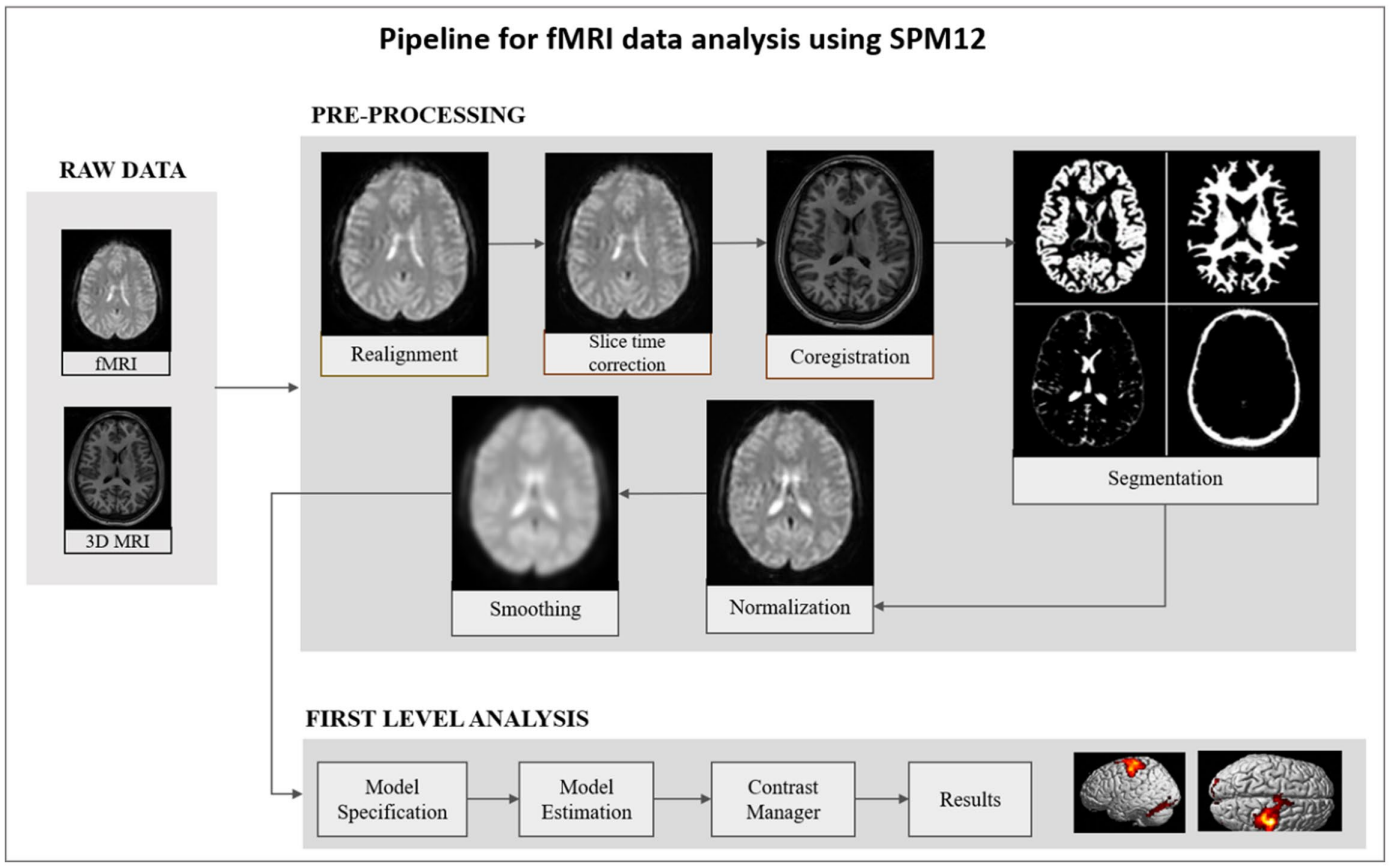
A schematic diagram consisting of the steps involved in the fMRI data processing pipeline. Raw data is pre-processed by realignment, slice time correction, co-registration, segmentation, normalization, and smoothing. The pre-processed smoothed image is the input for first level analysis. At subject level, general linear model was applied, and estimation was performed to obtain activation maps from the whole brain.

In the fMRI pipeline (Figure 3), individual raw images were slice time corrected and realigned to the mean functional image using six rigid body transformation parameters for head motion correction. To classify brain tissue, the anatomical T1-weighted MR image was co-registered with the mean functional image and segmented. The realigned and slice time corrected fMRI images were normalized with respect to the Montreal Neurological Institute (MNI) space brain atlas. Using a Gaussian kernel with a 6 mm full-width at half maximum (FWHM), the normalized functional image was spatially smoothed. A general linear model analysis (GLM) was applied to obtain whole brain activation (threshold of *p* < 0.01; uncorrected) maps for each subject. The analysis pipeline allows users to determine the threshold value. To specify a model, six head motion parameters and onset conditions as predictors were used. Convolution of the predictors with the canonical double gamma function and a temporal high pass filter (cutoff = 128 s) served as the basis for model estimation. The design matrix comprised of sessions corresponding to each run and regressors were defined for the active and baseline conditions. The pipeline also modeled linear residual movement effects by including motion regressors derived from rigid body realignment.

##### DWI data analysis pipeline

**Figure 4:**
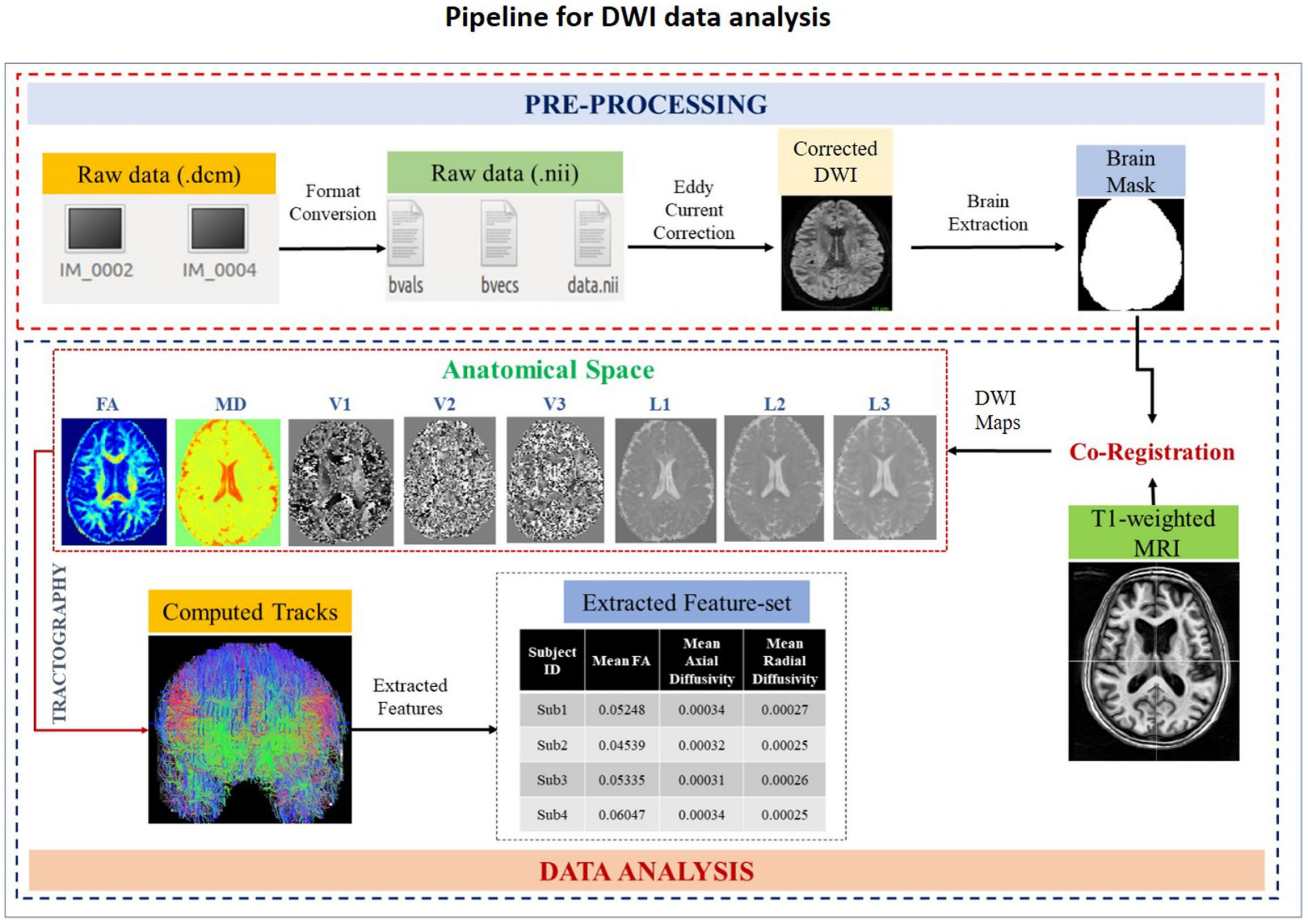
A schematic diagram depicting various steps of DWI data processing. The designed pipeline is composed of two steps: pre-processing, and data-analysis. The file format conversion, eddy current correction and brain extraction are the part of the pre-processing stage. Co-registration, DWI maps generation and extraction of features belong to the data analysis stage. After data analysis, an excel sheet containing a list of extracted features corresponding to all subjects is generated. FA, Fractional Anisotropy; MD, Mean Diffusivity; V1, 1st Eigen Vector; V2, 2nd Eigen Vector; V3, 3rd Eigen Vector; L1, 1st Eigen Values; L2, 2nd Eigen Value; L3, 3rd Eigen values.

MRI-based DWI measures the tissue integrity based on the diffusion of water in the brain. Pathological features (e.g., myelination, ischemia, inflammation) affecting microstructural composition in the brain can be measured using diffusion tensor imaging (DTI) technique (34). In SWADESH, the DTI data is analyzed using multiple steps as indicated in Figure 4. The pipeline consists of custom-made python-based scripts, developed using in-built functions available in the FSL-BET toolbox (35). Users can process DTI data using the customized pipeline in SWADESH platform, without installing additional software. This processing pipeline accepts only the NIfTI format of 4D DTI data input for processing, the raw data in DICOM format needs to be converted into NIfTI (.nii) format using dcm2niix for further processing. FSL FDT diffusion software^2^ was utilized for eddy current correction and brain mask generation. Later, the processed DTI image was co-registered with a T1-weighted MRI image to create a common template for alignment. Anatomical maps (i.e., eigenvectors, eigenvalues, mean diffusivity, and fractional anisotropy) were generated using DTIFIT^3^ on a co-registered image (36). The DTI features are extracted directly from the whole white matter. No region is specified in the available pipeline. Eventually, the features (e.g., mean of eigenvectors, mean of eigenvalues, mean diffusivity, the sum of squared error, relative anisotropy, and spherical diffusion variance) were extracted from the generated anatomical maps (37). The extracted set of features were presented in the excel sheet for further analysis purposes. The raw data utilized to represent the processing steps in Figure 4 was DWI data obtained from Philips Achieva 3 T MRI machine. The obtained results are shown corresponding to each step of processing. Tractography using Brain Suite package^4^ was also performed for visual analysis of computed tracts. The output of this processing pipeline is stored in a zip folder consisting of anatomical maps and excel sheet.

##### QSM data analysis pipeline

**Figure 5:**
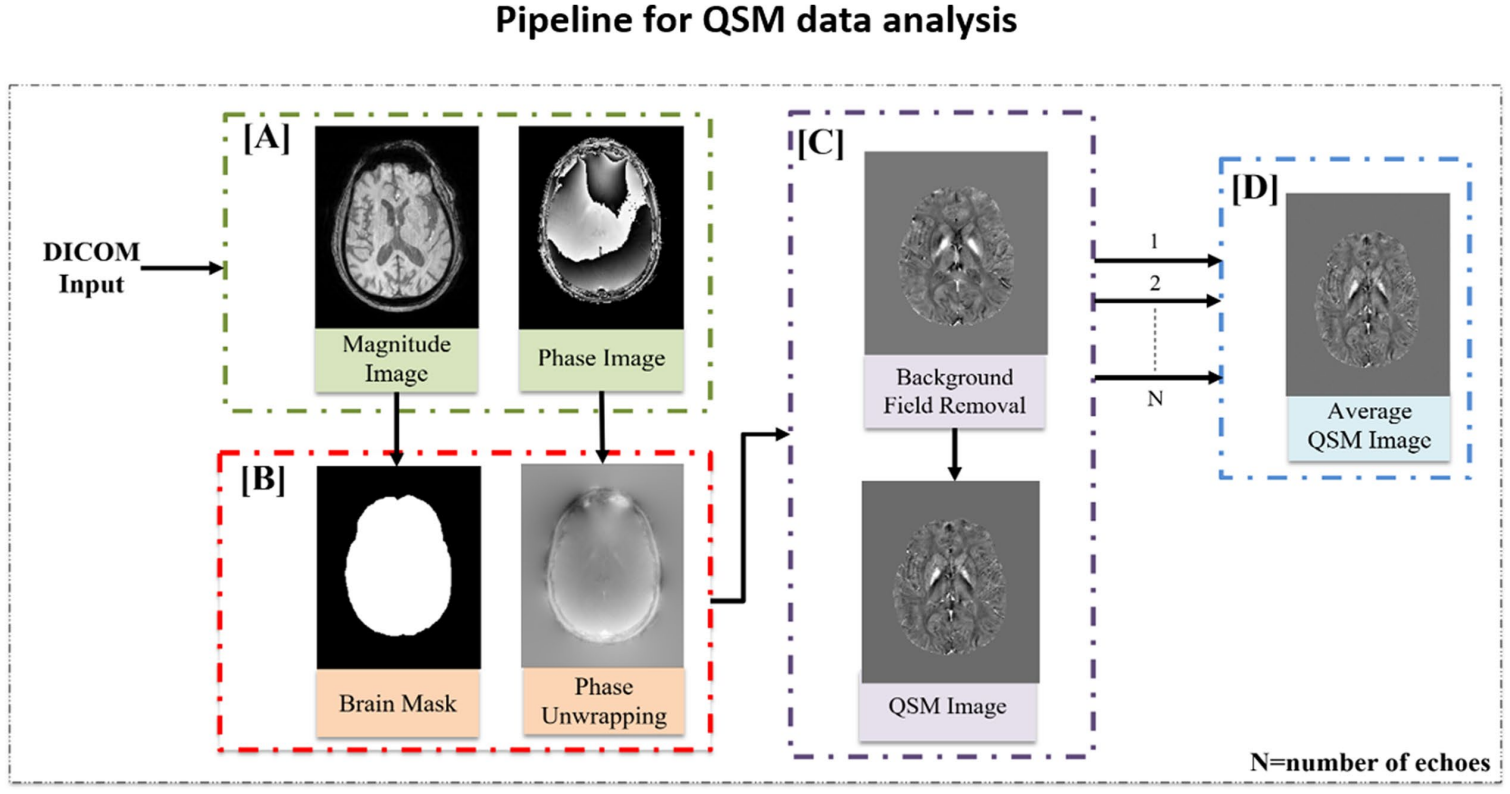
QSM data processing pipeline. **(A)** Magnitude and phase images were extracted from the DICOM file. **(B)** Binary mask was created using the magnitude image followed by phase unwrapping using the Laplacian phase unwrapping method. **(C)** Background field removal was performed using the V-SHARP method followed by susceptibility inversion using iLSQR technique to extract the QSM image. **(D)** Average QSM image was computed by averaging the susceptibility map obtained for each echo.

QSM is an advanced MR technique used to determine the degree of susceptibility in brain tissue. It gives an indirect measure of metals (iron, copper, calcium, etc.) in the specific brain region (38). QSM data processing involves a series of sophisticated processing steps that are based on understanding the relationship between magnetic susceptibility, magnetic field, and modulated MR signal. A pipeline of MATLAB-based scripts was developed to automate QSM data analysis (Figure 5) (39). In the customized pipeline, phase and magnitude images were extracted from the DICOM input image and a binary mask was created using FSL’s brain extraction toolbox (BET) (35). To unwrap the raw phase image, a Laplacian-based algorithm was applied (40), and the background field was removed with the help of brain masks using the V-SHARP method (Variable Sophisticated Harmonic Artifact Reduction for Phase data) (41). Iterative Least Square (iLSQR) was used to process each echo separately to calculate the quantitative susceptibility maps (42). To calculate the average QSM image, the susceptibility maps obtained for each echo were averaged.

##### Neuropsychological data analysis

**Figure 6:**
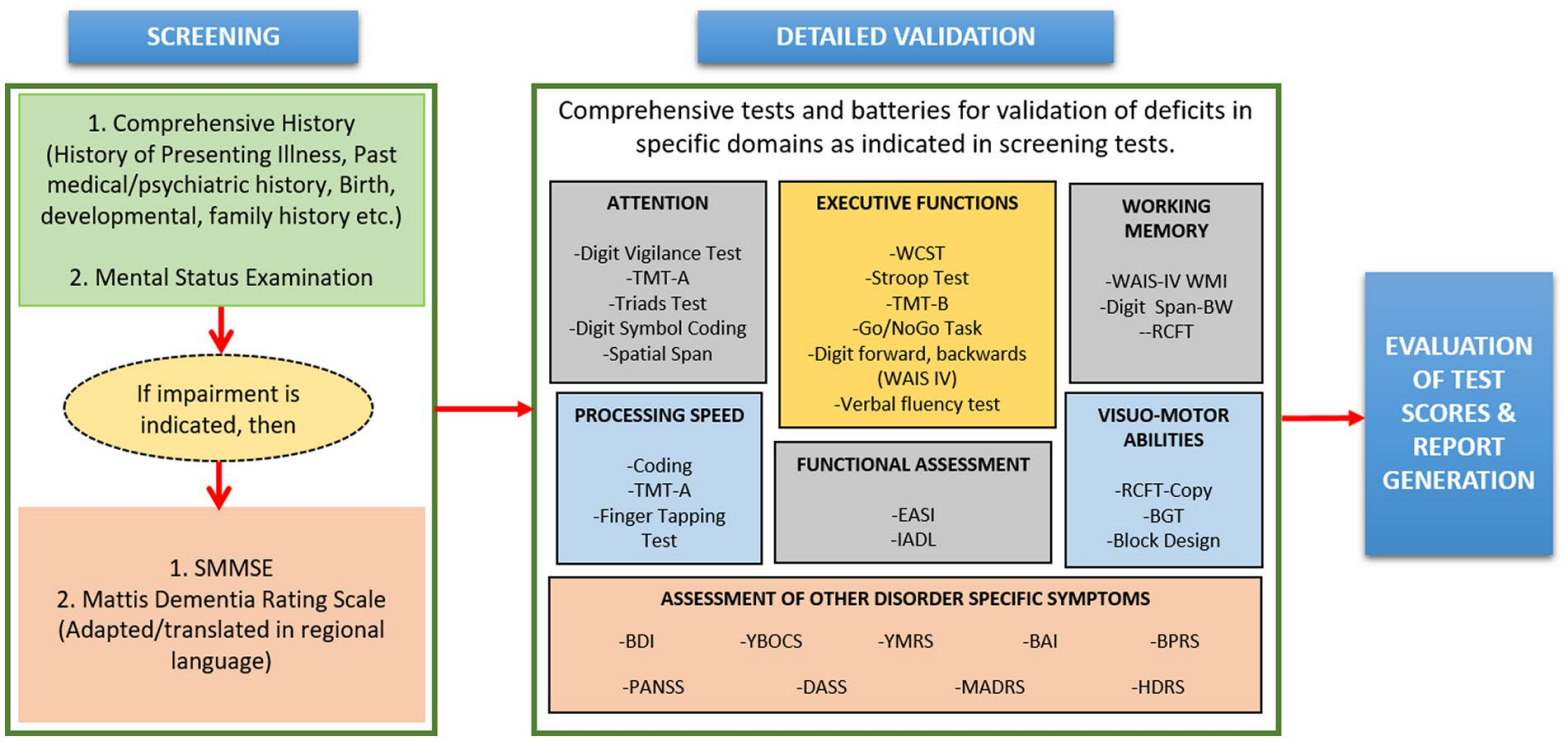
A schematic diagram of the steps involved in the neuropsychological data analytical pipeline. Screening of individuals includes various steps like mental status examination to determine cognitive behavior. Further, comprehensive test batteries were performed for detailed validation. Finally, the obtained information from comprehensive tests was utilized for assessment of treatment responses, interpretation about diseases and different applications. SMMSE, standardized mini-mental state examination; TMT, trail making test; WCST, Wisconsin Card Sorting Test; WAIS-IV, Wechsler Adult Intelligence Scale, Fourth Edition; RCFT, Rey Complex Figure-Test; EASI, Early Assessment Self Inventory; IADL, Instrumental Activities of Daily Living, BW: Backward; BGT, Bender-Gestalt test; BDI, Beck Depression Inventory; YBOCS, Yale-Brown Obsessive-Compulsive Scale; YMRS, Young Mania Rating Scale; BAI, Beck Anxiety Inventory; BPRS, Brief Psychiatric Rating Scale; PANSS, Positive and Negative Syndrome Scale; DASS, Depression, Anxiety and Stress Scale; MADRS, Montgomery and Asberg Depression Rating Scale; HDRS, Hamilton Depression Rating Scale.

Neuropsychological data contains the behavioral signature of brain dysfunction (43), derived by standardized tests, behavioral observations, and qualitative indicators. For neuropsychological tests, the individual is interviewed, and a thorough history is elicited from them and their family members. This includes developmental history, family history, history of present psychiatric/neurodegenerative illness as well as that of past illnesses. This is followed by a comprehensive mental status examination (MSE) (44). The MSE assesses all relevant areas of mental functioning such as speech, motor activity, mood, affect, thought-content, executive functioning, intelligence, abstraction, memory, language; judgment and insight into the illness (45). At the same time, severity rating scales such as Beck Depression Inventory-II, Positive and Negative Syndrome Scale, Young Mania Rating Scale etc. are applied to assess the severity of the symptoms of psychiatric disorder (if present). If during the MSE, impairments in cognitive functioning are observed, the individual is screened for cognitive deficits using instruments such as the Standardized Mini Mental State Examination (SMMSE) (46) or Montreal Cognitive Assessment (MoCA) (47). If on these tests, cognitive dysfunction is indicated, the individual is subjected to comprehensive neuropsychological evaluation using standardized batteries and tests, which are presented in Figure 6. In SWADESH, the neuropsychological data was collected for various classes *viz.* HY, HO, MCI, AD and PD. Various neuropsychological tests as described above were administered to assess cognitive functioning. All the data was analyzed, and the results were accepted, and outlier data were stored in excel sheet in the database.

#### GUI-based data analytical pipeline

##### KALPANA-MRS data processing tool

MRS is a non-invasive MR based modality to determine various metabolites in the brain and other organs. It is used to obtain *in vivo* concentration measures of specific chemicals like N-acetyl aspartate (NAA), myo-Inositol (mI), creatine (Cr), choline (Cho), gamma aminobutyric acid (GABA), glutamate, glutamine and, glutathione (GSH) (48). Spectral editing techniques like MEshcher–GArwood Point RESolved Spectroscopy (MEGA-PRESS) (49, 50) allow suppression of unwanted metabolite signals and desired peaks are detected without any ambiguity. Usually, single voxel spectroscopy (SVS) is preferred due to its ability to generate a high-quality spectrum with excellent shimming and high signal to noise ratios (SNR) (51, 52). MRS data needs to be inspected carefully and processed by a series of steps and, therefore it cannot be fully automated. In the platform, KALPANA, a dedicated package for MRS processing is provided to users upon request (53). It is a MATLAB-based in-house developed MRS data processing pipeline utilized for quantifying the metabolites concentration. Components of the popular SPM12 tools (54) have also been integrated with the KALPANA package for MRI segmentation and normalization.

**Figure 7:**
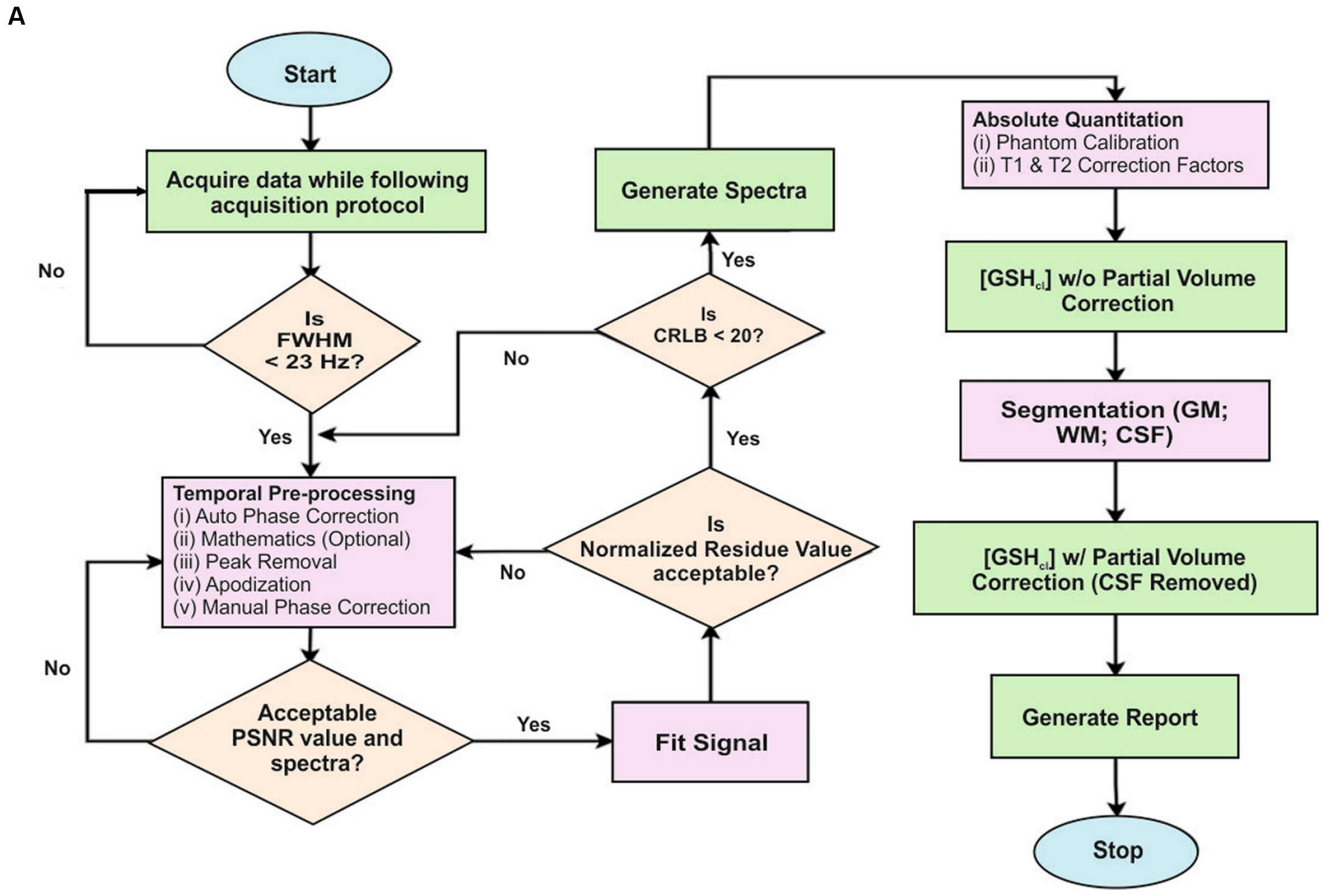

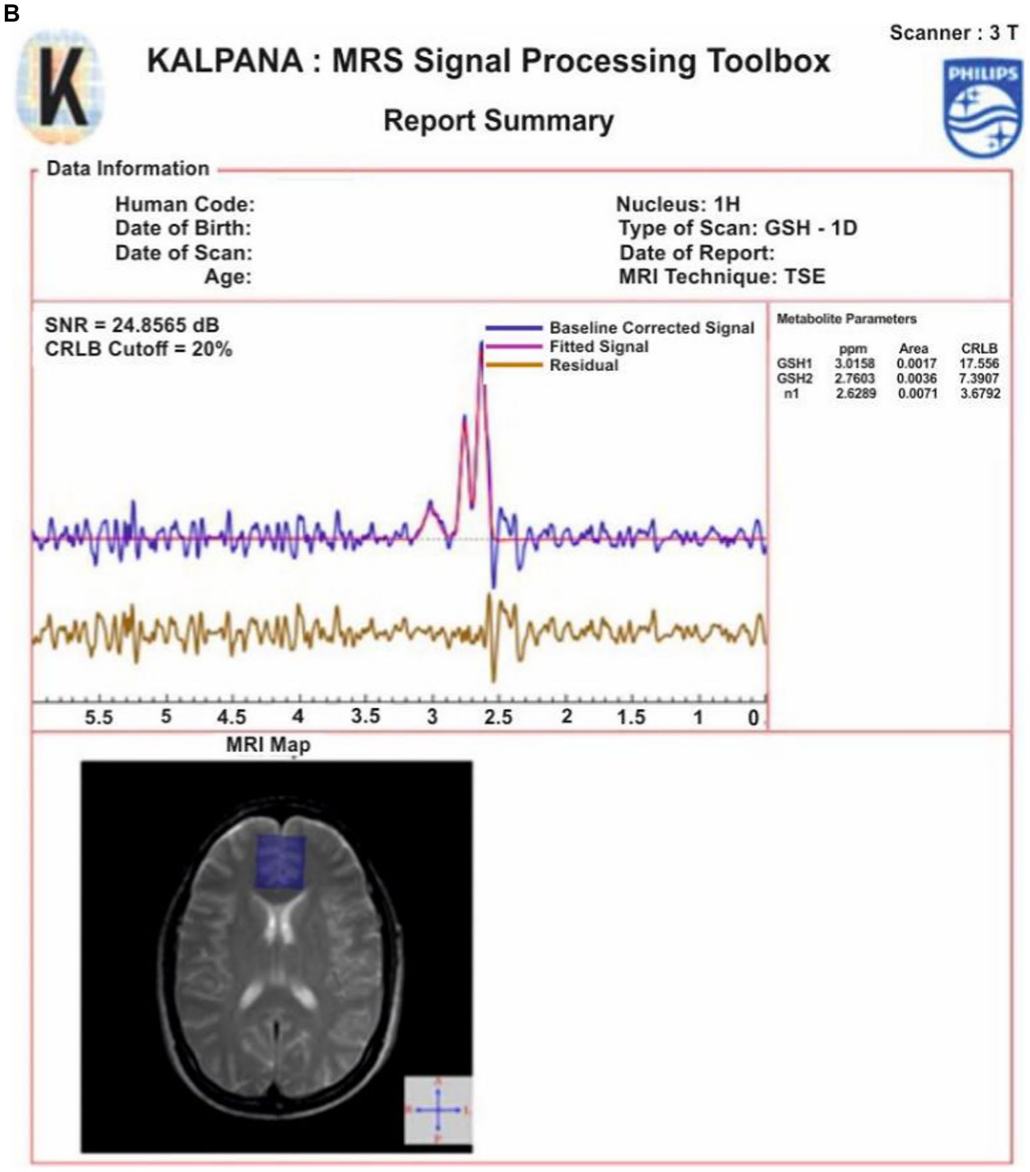
**(A)** A flowchart describing the steps for MRS data processing using KALPANA package. First, the acquired raw data is temporally pre-processed. Later, PSNR value is calculated to quantify the quality of spectra. A quality checked spectrum is then fitted for further processing. Furthermore, normalized residue and CRLB are calculated. When both the criteria are fulfilled, the final report is generated subsequently. **(B)** Processing and peak fitting of GSH data using KALPANA package.

The flow diagram for the MRS data processing for quantification of GSH in KALPANA is presented in Figure 7A. For an explanation of various steps in the KALPANA package, MRS data of a subject is considered that had been acquired with time of echo (TE) of 120/130 ms by placing a single voxel of dimension 25 × 25 × 25 mm^3^. After loading the signal in KALPANA, temporal pre-processing is performed wherein the mathematics is performed over a set of ON and OFF spectra of frequency selective editing pulse. The water peaks removal is carried out using Hankel Lanczos singular value deposition filter (55). Further, the signal is phase corrected to restore pure absorption spectra. Apodization is then performed for further smoothening of the signal where two functions, Exponential and Gaussian are applied collectively on the signal.

It has been reported in the existing literature that apodization should be limited to 2 Hz otherwise significant amount of data is lost due to over smoothening of the signal (56). The apodized MRS signal is baseline corrected using one of the baseline correction methods among singular spectrum analysis (SSA), spline, and wavelet. We have applied the SSA method of baseline correction. Time-domain and frequency-domain curve fitting procedure is also applied for the quantitation of metabolites. The area under the curve of a particular metabolite, Peak SNR (PSNR), Cramér Rao lower bounds (CRLB), and concentration of the metabolites are then computed from the fitted signal of metabolites and checked for their acceptance. Further, phantom calibration followed with T1 and T2 correction factors is performed for absolute quantitation of metabolites. Since, it has been reported that the concentration of metabolites is negligible in cerebral spinal fluid (CSF) (57) and only gray matter (GM) and white matter (WM) brain tissues contain metabolites. This partial volume correction (PVC) was applied. For PVC, the brain tissues are segmented using SPM12 and the metabolite concentration. Concentration in the brain is corrected for CSF contamination using PVC factor to obtain absolute quantitation of metabolites in the brain (57). Following these steps, results are exported, and the final report is generated (Figure 7B).

### SWADESH web-portal

SWADESH web portal was developed to provide a comprehensive and user-friendly platform for accessing and processing diverse neuroimaging data corresponding to various modalities namely MRI, fMRI, DTI, QSM, MRS, and neuropsychological data. The link to access the SWADESH portal is: http://swadesh.nbrc.res.in/.

#### Computation and technology used

SWADESH web application begins with the load balancer receiving requests from the users. The load balancer then assigns individual server nodes to each client request. The allocation of resources is based on existing server load that is being monitored by the load balancer. The servers run services or back-end processes that access data on shared external storage disks. Client nodes access the front-end of the web-application. A client system then sends a request from the SWADESH web application to the servers for accessing various analysis pipelines or to request data from the anonymized data repository hosted on SWADESH. The server then processes the request and displays the results to the user.

Server overloading is the most common issue which can occur due to sudden natural traffic spikes. Malware attacks can also be a reason for server overload by generating massive number of requests to overflow the servers. To avoid server overloading, load balancing has been deployed on the SWADESH servers. A load balancer lies between the clients and servers, wherein the traffic on a network is distributed across a group of servers (58). Rate limiters have also been configured on the load balancer to prevent server overloading.

The SWADESH web portal consists of front-end and back-end designs as described in the following sections.

#### Front-end

**Figure 8:**
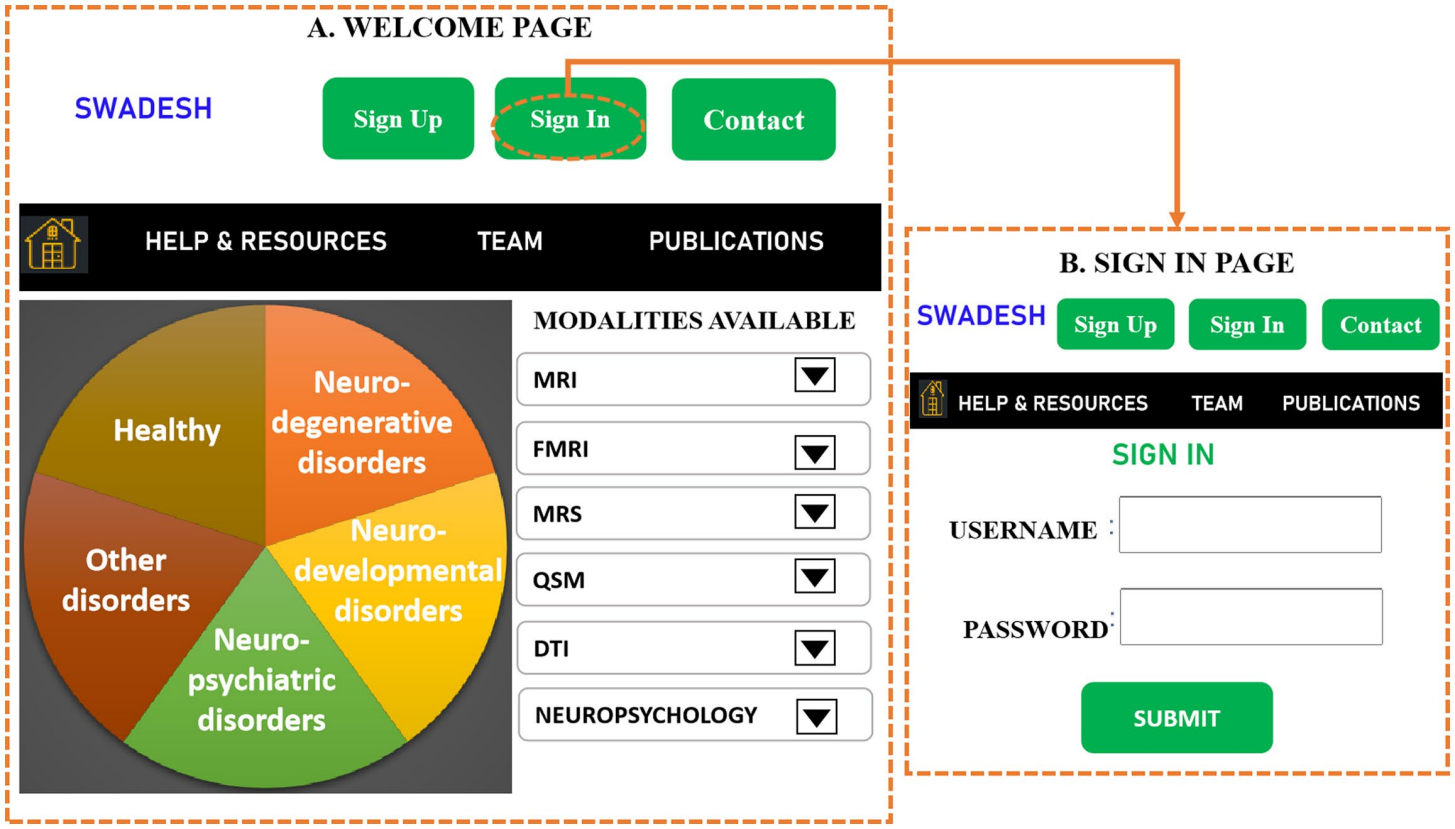

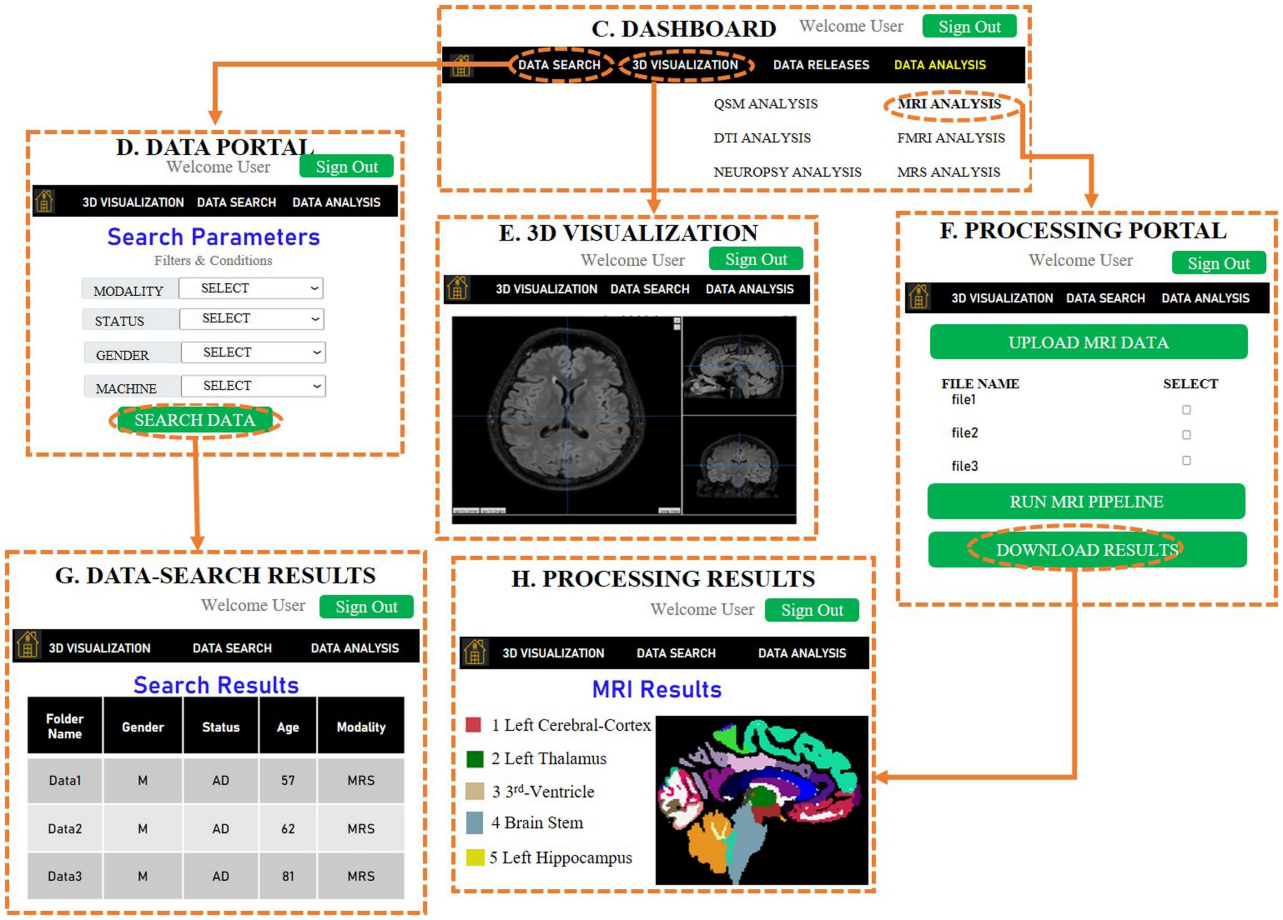
A representative sketch of web-based SWADESH system, consisting of various stages. **(A)** Front-page provides the introduction of SWADESH webpage, **(B)** In the second stage, the user can sign in and sign up for accessibility of SWADESH, **(C)** After signing in, the third page provides the dashboard to the user along with options of data search, visualize and analyze multimodal neuroimaging data, **(D)** A sub-page of the third page provides the user option to search data corresponding to various modalities, status, gender and scanner type (Philips, Siemens etc.), **(E)** image viewer to display the three-dimensional picture of the image to perform a visual inspection of image quality. **(F)** Processing portal for running modality-specific pipeline for the data to be analyzed. (For demonstration here, MRI pipeline is selected), **(G)** The web-page of data search results as per specified parameters in **(D)**, **(H)** Processing results obtained after running the modality-specific analytic pipeline on uploaded data in **(F)**. At present, the platform hosts neuroimaging data of HY, HO, and patients with AD, MCI, and PD. To provide a generic approach in the future, we represent neuroimaging data across five modules, namely neurodegenerative, neuropsychiatric, neurodevelopmental, other disorders, and healthy subjects as depicted in part **(A)**.

The SWADESH platform can be accessed for obtaining the data corresponding to various modalities namely, MRI, fMRI, DTI, QSM and neuropsychological data. The developed pipelines are utilized to perform modality specific analysis and then user can draw inferences. The pipeline followed for each modality is provided in the previous sections of this manuscript. The processing pipelines of each of the modalities (except MRS) are developed utilizing Python 3.7. MRS data can be processed efficiently using the KALPANA package (53). The web-based SWADESH system is depicted in Figure 8. The introductory page of SWADESH contains an overview for operating the website along with a count for available data corresponding to each modality. The front page is followed by a sign-in page, to register or sign-in to the SWADESH portal. After sign-in, user can access and download the data and processing pipeline separately. On the data portal, the user can download the required data considering meta data such as modality, age, and gender. On the processing portal, the user can access modality-wise pages to upload image data, process pipeline and then download the results of respective modality. Once the neuroimaging or neuropsychological data is uploaded and sent for analysis, the automated pipeline of the corresponding modality gets activated, and generates the outcome in the results folder.

SWADESH is structured in such a way that if any user does not want to analyze the data (taken from the SWADESH portal) using our pipeline, then they can just download the data and use their own analysis pipeline. Also, if the user wants to analyze data collected from multiple sources (outside the SWADESH database) and just wants to use our processing pipelines, then the user needs to compile the data in one folder and upload it to our data analysis portal. Another reason for these two platforms (database and analytics) to be kept different is that these analysis pipelines include only basic pre-processing scripts arranged for operation in a seamless fashion. Therefore, if any user wants to perform extra preprocessing or noise removal steps, they can do so before processing the data using our portal.

#### Back-end

The backend part of the SWADESH web portal consists of a DJANGO-based framework (59) and has been developed on Ubuntu *20.04.2 LTS* system. The server infrastructure consists of *11* servers with *2 X Intel Xeon Gold 6,248, 2.5G, 20C/40 T, 10.4GT/s, 27.5 M* cache, and turbo processor. One of the servers is being used solely as the data repository which consists of all the neuroimaging data which have been segregated according to the search algorithms integrated into the SWADESH. It also hosts individual user data for situations where the user can upload data the data processing pipelines. All the servers in the network access the data server via *Network File Sharing (NFS)*. It allows the other servers to act as a client to individually mount the data server on their operating systems. The data server also hosts the scripts that run the analysis procedures as well as provide the results files. One server is deployed as the load balancer. The rest nine servers relate to each other for handling data searching, processing, and analysis. Each server is connected via a 1 Gbps internet connection to ensure fast upload and download speeds.

In SWADESH, NGINX has been used to handle load balancing to reduce the load on a single server and to improve system performance (60). NGINX is presently using the round-robin algorithm for performing load balancing on the client requests (61). Round Robin is the load balancing technique which is being used to run through the list of upstream servers in sequence assigning the next connection request to each one in turn. Each server is selected as per the sequence defined in the *load*-*balancer configuration* file and balances the number of requests equally for short operations. Load balancing is being performed by NGINX (Engine X) which has been configured on one of the servers. The Django setting file has been configured to accept requests from only one IP address which is of the server. All the requests are handled by NGINX which considers the pre-existing load on the servers and directs the requests.

The NGINX configuration files have also been modified to allow individual request handling for a duration of 24 hours after which a timeout will be issued. Each user is also allowed to have a maximum upload of 23 GB to ensure maximum efficiency. A timeout window of 24 hours has been provided keeping the average MRI processing time (approximately 7 hours per batch) into consideration. Each batch can handle *96* images at one time (individual session).

#### Web security

Security is the main concern while developing a web application for protecting the user account information and data from unauthorized access, and destruction, or disruption. Django provides several security features such as cross site scripting (XSS) protection, cross site request forgery (CSRF) protection, SQL injection protection, clickjacking protection, and host header validation (62). The Django template has inbuilt mechanisms to defend against the majority of XSS attacks which involve injecting malicious scripts into trusted websites (63). Furthermore, Django provides protection against CSRF attacks by declining the requests which are not authorized (64). SQL injection protection shields the data leakage or deletion of records from the SQL platform (65). The clickjacking protection provided by Django prevents the site from being trapped in third-party sites (66).

## Results

### Data search

**Figure 9:**
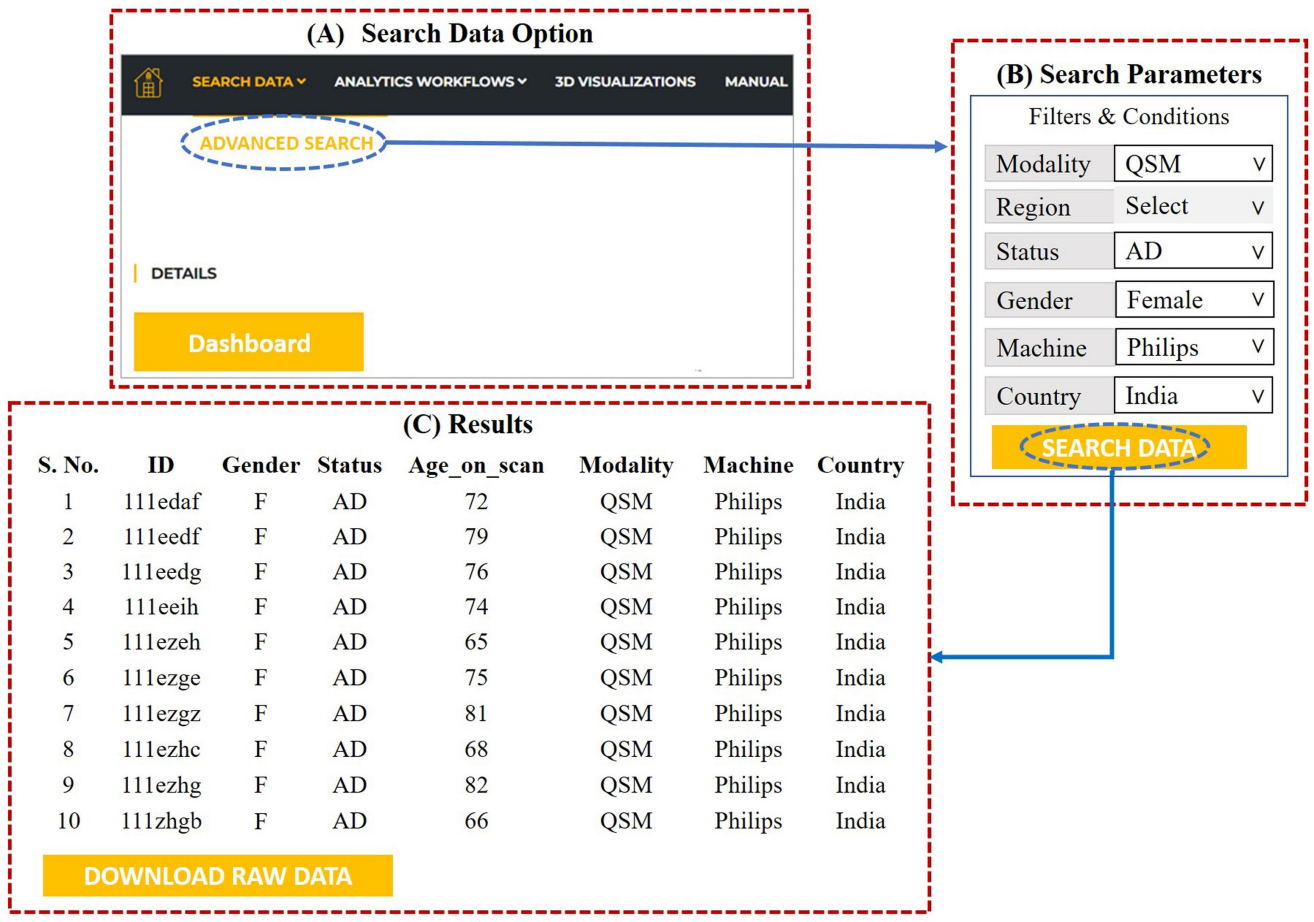
**(A)** Data search option, **(B)** advanced search dialog for specifying the search parameters, **(C)** results as per the specifications provided by the user.

Using the “Search Data” option (please refer to Figure 9A), user can access the multimodal neuroimaging and behavioral data for various brain disorders. The user needs to click on the “Advanced Search” option under the “Search Data” to specify the filters and conditions of the required data. This is depicted in Figure 9B. The specifications’ options are: type of modality (MRI, fMRI, QSM, MRS, DTI, Neuropsychological), gender (male, female), status (AD, MCI, HO, PD), scanner (Philips, GE, and Siemens) and country. The region option has various anatomical area for MRS study.

After specifying the parameters, invoking the “search data” at the bottom gives access to the required data. The user can then download the specified data by clicking on “Download Raw Data” (Figure 9C).

### 3D visualizations

**Figure 10:**
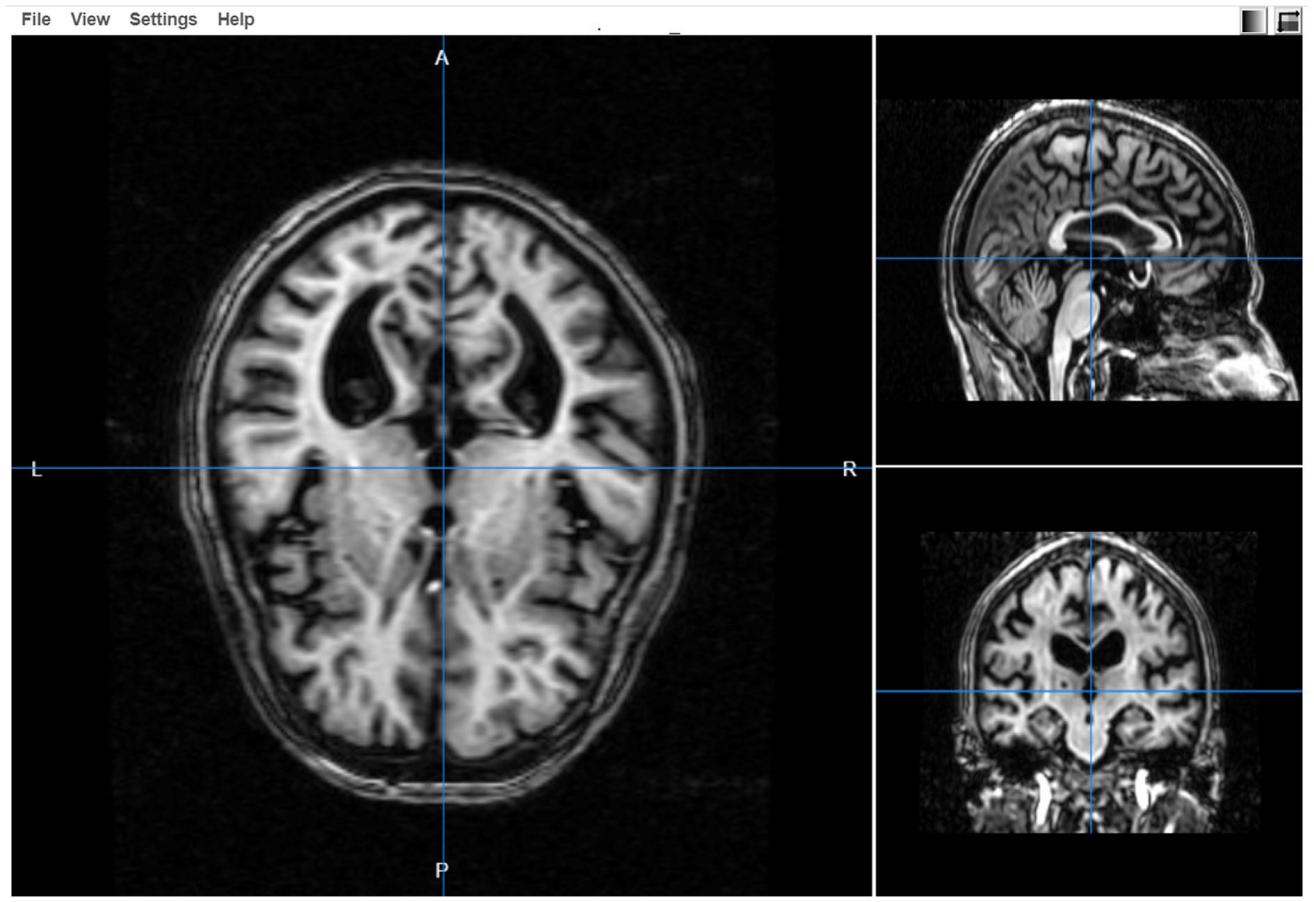
Three-dimensional view of the selected file in the SWADESH image viewer.

The 3-dimensional image viewer is also provided in the SWADESH platform. Using the “3D Visualizations” option on the dashboard, the user can view an MRI image by selecting the “file option” and specifying the file. Once the image is selected, the image viewer helps to review the axial, sagittal and coronal images (see Figure 10).

### Analytic workflows

**Figure 11:**
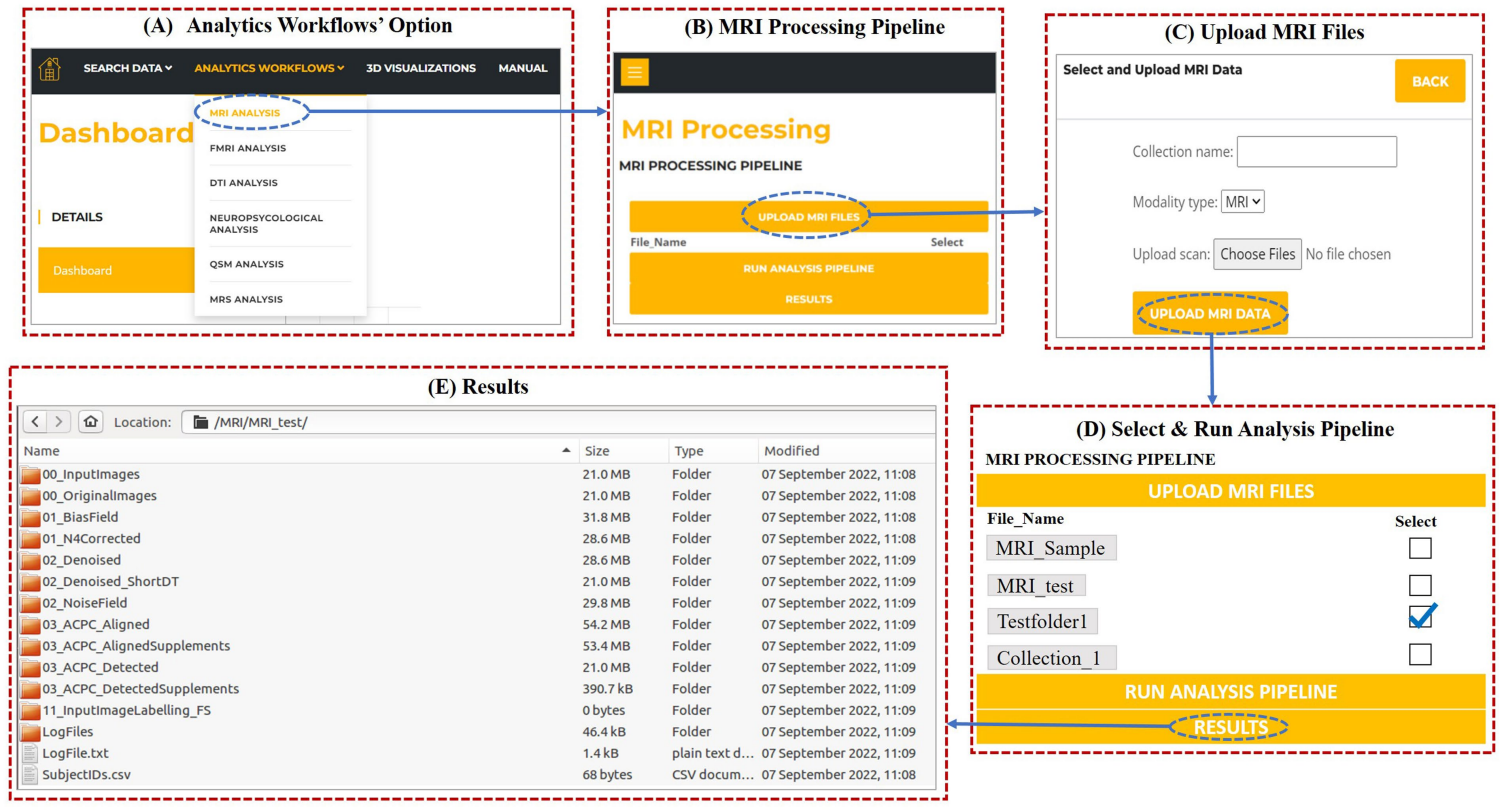
**(A)** Analytics Workflows option on the dashboard to process modality-wise data. **(B)** MRI Analysis dialog on selecting MRS analysis under “Analytics Workflows.” **(C)** Pop-up dialog after clicking “Upload MRI files.” **(D)** MRI Analysis window for processing MRI data. **(E)** Processed files obtained after running the MRI processing pipeline.

In SWADESH, the data analytical tools are available to process MRI, fMRI, DTI, QSM and Neuropsychological scores. The user can use the option of “Analytics Workflows” on the dashboard to process various types of neuroimaging data. By clicking on the “Analytics Workflows,” user can select the analytical pipelines for various modalities (Figure 11A).

For demonstration, we are showing the use of MRI analytical pipeline. From the drop-down list under “Analytic Workflows,” user can select MRI Analysis. When the user clicks on “MRI analysis,” a window as shown in Figure 11B opens, where the user can upload the MRI data to be processed and download the results. On clicking “Upload MRI files,” user can name the collection (e.g., “MRI_test”), select and upload the MRI files for processing (Figure 11C).

Once the data gets uploaded, the user needs to select (tick) the collection name for running the MRI analytics pipeline on the selected collection. Then, the user can invoke “Run analysis pipeline,” to run the processing script as shown in Figure 11D.

Once the processing is complete, user can invoke the “Results” button to download the results. On clicking the results button, a zip folder is downloaded that contains all the processed files. For a particular collection, the processed files of MRI contain the label generated files as shown in Figure 11E. In a similar way, users can upload the data and process it for other neuroimaging analytic pipelines. Due to space constraints, other results from other modalities’ pipelines can be found in the user manual (please see Supplementary material).

## Discussion

The neuroimaging community is creating resources for data analytics including software, libraries, scripts, and pipelines or workflows. Efforts have been made to integrate these resources on a single platform. A cloud computing system is capable of providing unlimited and scalable computing resources but difficult to integrate because it requires specialized knowledge. With the help of web interfaces, cloud computing can be made accessible so that researchers in specific domains can take advantage of its full potential. Many large-scale neuroimaging data and analytics platforms have been launched in recent years with the Canadian Brain Imaging Research Platform (CBRAIN) (67), heterogeneous data sources and compute grids can be combined into one, secure web interface. Another platform called BrainLife (68) offers cloud computing resources and data sharing services. Similarly, data management and analysis platform Brain-Code aims to encourage collaboration and discovery across different brain disorders (69). These platforms host a wide range of neuroimaging data from various modalities. Project SWADESH aims to include neurochemical data using MRS, magnetic susceptibility data using QSM as well as modality-specific analytical pipelines along with neuroimaging and behavioral data. Additionally, the project is designed to provide and expand data, analytical pipelines for academic use, to facilitate the integration of multisite, multi-disease data for collaborative research globally. This initiative has the distinction of being the first scalable multimodal neuroimaging database with data analytics that provides neuroimaging data from the Indian population.

SWADESH is a secure and convenient web-based system that includes rigorous analytical pipelines for the assessment of multi-modal neuroimage data, namely MRI, DTI, fMRI, QSM along with neuropsychological data. Large volumes of data are integrated within SWADESH to support scientific questions and analytics across multiple brain disorders and modalities. This project is an effort to build and enrich a multidimensional neuroimaging platform for identifying early diagnostic biomarkers of various brain diseases. Presently, SWADESH database contains data collected more than 200 patients with AD, MCI, and PD. The platform also includes more than 210 healthy old individuals and more than 550 healthy young as controls. As neuroimaging data and new analytical tools are added constantly to the platform, the platform’s capabilities are being expanded. The project features computational pipelines for multimodal neuroimaging data. One of the key goals of SWADESH is to build a neuroimaging big data architecture that analyses and manages five domains namely neurodegenerative (such as MCI, AD, and PD); neuropsychiatric (such as schizophrenia and bipolar disorder); neurodevelopmental (such as autism and epilepsy); other disorders and healthy subjects.

The computational infrastructure for SWADESH is implemented and maintained by the Neuroimaging and Neurospectroscopy (NINS) Lab at the National Brain Research Centre (NBRC), India.^5^ Currently, SWADESH contains data only from the Philips machine (3T), however, data from Siemens scanner (3T) are being collected and will be uploaded. In future, data from GE scanner will also be uploaded. At present, the platform is supported by eleven servers. To increase SWADESH’s load handling capacity, more servers are being added. For improved performance and speed, the current uplink and downlink speed of 1Gbps will be upgraded to 10 Gbps in the future. Since the existing infrastructure has limited storage capacity, certain steps have been taken to limit data uploads, such as limiting the amount of data that can be uploaded per session to 23 GB. In addition, to maintain the efficiency of the system, data kept longer than a fortnight is automatically removed. Data cleaning capabilities will be provided to user through dedicated servers for storing redundant data in the future.

In the initial phase, project SWADESH aims to integrate neuroimaging data generated through multimodal neuroimaging and neuropsychological assessment at NBRC, India, and its collaborating institutes. This centralized platform for multimodal neuroimaging data and analysis supports the management, sharing, and analysis of multidimensional neuroimaging data, making SWADESH a great opportunity not only to strengthen and expand these collaborations within India as well across international neuroscience communities. SWADESH is primarily developed for neuroimaging researchers, neuroradiologists, and will have profound implications for medical research. We have already developed a novel platform called “GANGOTRI” for the quality check of various imaging data (manuscript under review).

### Data privacy

Data privacy of each participant is paramount for us. All personal information of the participants in the database are completely anonymized and rechecked. Data are available from the database upon request and subsequent review.

## Data availability statement

The raw data supporting the conclusions of this article will be made available by the authors, without undue reservation.

## Ethics statement

All studies involving humans were approved by NBRC Human Ethics Committee. The studies were conducted in accordance with the local legislation and institutional requirements. The participants provided their written informed consent to participate in those studies.

## Author contributions

PM: Conceptualization, Formal analysis, Funding acquisition, Investigation, Validation, Writing – original draft, Writing – review & editing, Methodology, Supervision. KJ: Data curation, Methodology, Software, Validation, Writing – review & editing. SR: Data curation, Methodology, Software, Writing – review & editing. YA: Data curation, Investigation, Methodology, Software, Validation, Visualization, Writing – review & editing. ShS: Data curation, Methodology, Software, Validation, Writing – review & editing. SJ: Data curation, Investigation, Methodology, Writing – review & editing. AG: Data curation, Methodology, Software, Writing – review & editing. ZA: Data curation, Methodology, Software, Writing – review & editing. JM: Validation, Writing – review & editing. KSi: Data curation, Software, Writing – review & editing. KSa: Data curation, Writing – review & editing. MT: Data curation, Resources, Writing – review & editing. PS: Data curation, Resources, Writing – review & editing. AS: Data curation, Writing – review & editing. SG: Data curation, Writing – review & editing. SaS: Resources, Writing – review & editing.

## Footnotes

^1^ https://github.com/rii-mango/Papaya

^2^ https://fsl.fmrib.ox.ac.uk/fsl/fslwiki/FDT

^3^ https://users.fmrib.ox.ac.uk/~behrens/fdt_docs/fdt_dtifit.html

^4^ http://brainsuite.org/

^5^ http://www.nbrc.ac.in/newweb/

## Abbreviations

AC: Anterior commissure
AD: Alzheimer’s disease
DTI: Diffusion tensor imaging
DWI: Diffusion Weighted Imaging
fMRI: Functional Magnetic Resonance Imaging
HY: Healthy young
HO: Healthy old
MCI: Mild cognitive impairment
MRI: Magnetic resonance imaging
MRS: Magnetic resonance spectroscopy
NIfTI: Neuroimaging Informatics Technology Initiative
PC: Posterior commissure
PD: Parkinson’s disease
QSM: Quantitative susceptibility mapping
