# Supplementary material for: SWADESH: a multimodal multi-disease brain imaging and neuropsychological database and data analytics platform
Source: Front Neurol. 2023 Oct 4;14:1258116. doi: 10.3389/fneur.2023.1258116 (PMC10582723; doi:10.3389/fneur.2023.1258116)
Supplement: Supplementary file 2 [file Data_Sheet_1.pdf]

SWADESH

Version: 2.0

September, 2023

Multimodal Neuroimaging and Behavioral Data and Data Analytics

(A NINS Creation)

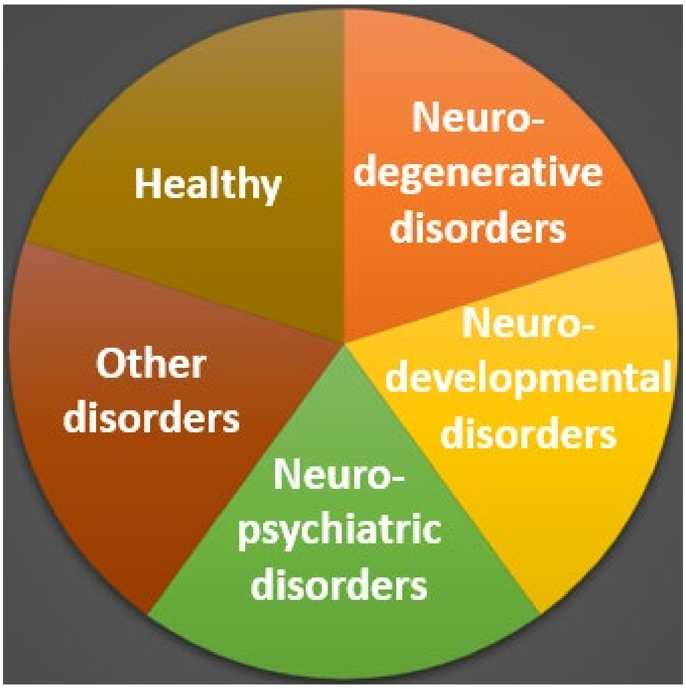

User Manual

Neuroimaging and Neurospectroscopy (NINS) Laboratory
National Brain Research Centre (NBRC), Manesar
Gurgaon, Haryana, India

Contact us at: swadesh.nins@gmail.com

Supplementary material and website maintained by: Dr. Yashika Arora & Mr. Saurav Roy

Contents

1. Introduction 2
I. About 2
II. Aspects & Features 3
III. Overview of the functionalities 4
2. Getting started 6
3. Data Search 8
4. 3D Visualizations 10
5. Analytics Workflows 11
6. Additional Features 16
7. Help and technical support 16
8. Citation 16
9. Data Specifications 16

1. Introduction

I. About

SWADESH is a web-based platform for hosting large-scale multimodal neuroimaging, quality checked data with a big data architecture and analytical tools for various brain disorders. SWADESH is being developed under the supervision of Professor Pravat K Mandal, Scientist VII, at the Neuroimaging and Neurospectroscopy (NINS) Laboratory, National Brain Research Centre (NBRC), Manesar, India. SWADESH is a user-friendly platform that requires no computer programming experience. In order to use this platform, you simply need a web browser (a recent version of Firefox, Safari, Chrome or, Internet Explorer) and you don't need to install any special software. This manual provides an overview of the usage of SWADESH. It will describe the examples of typical data processes, from searching for multimodal neuroimaging data to running tasks and downloading results.

II. Aspects & Features

SWADESH is a fully featured system that supports multi-site neuroimaging clinical data along with in-house developed multimodal data analytical tools and AI-powered early diagnosis system.

a. Storage, Accessing, and Visualization

SWADESH is a searchable online repository for the neuroimaging and neuropsychological data, designed to serve a variety of functions. The whole data is organized as per the modality, along with meta information (such as disease status, gender, age, anatomical region, protocol followed during data acquisition etc.) required for describing the data. By applying filtering bar on the data in terms of modality, gender, and disease status, the user can search and sort the data to download the data of interest. Here, the MRI and fMRI data in NIFTI, PAR and REC format, QSM and DTI data in DICOM and MRS data in SPAR and SDAT format are available. Currently, we are having data only from the Philips MRI machine, however, data in other formats from other MRI machines (like General Electric (GE) and Siemens), will also be uploaded during expansion of this database.

SWADESH is designed with the idea that once the data is retrieved, it could be easily organized, visualized, processed, and analyzed. Data visualization is an important aspect in the analysis of neuroimaging data because sometimes key information can be accumulated from the data just by seeing it. For example, the quality of data can be determined on the basis of visual analysis. In this context, we deployed a pure JavaScript based medical research image viewer named as Papaya (https://rii-mango.github.io/Papaya/), supporting DICOM and NIFTI formats. This orthogonal viewer is configurable with many display, menu and control options and also compatible across a range of web browsers. Therefore, it can be run on a web server or as a local, shareable file.

b. Computing

SWADESH execution servers typically run in a regular user account on a server node. Server nodes run services or back-end applications that access data on the shared external disks. Client

nodes run front-end applications that retrieve data from the services provided by the server nodes. A client node receives requests from the SWADESH portal containing information about the requesting user, the location of specific data, tools and analytical pipelines, based on which the results are stored. The server then synchronizes the data and make preparation required by tools to successfully perform the requested tasks through this platform.

Server overloading is the most common issue while computing through such platforms. There are several common causes of server overload. Sudden natural traffic spikes when too many users attempt to use a site at once. Due to which a server can crash or cause server overload. Unavailability of servers due to sudden malfunction, hacking, or even planned maintenance. The backup server handles all extra traffic at such time and can easily experience server overload. Malware due to viruses can also be a reason for server overload by disrupting normal operations and causing abnormally high server traffic.

c. Security

Django is a high-level python-based framework for website development. During website development, security of user-data is a major concern. Therefore, Django provides following security features:

1. Cross site scripting (XSS) protection: XSS attacks allow a user to inject client-side scripts into the browsers of other users. Therefore, any malicious script can be stored in the database, whenever data is not sufficiently sanitized before inputting in a page. Django provides protection against majority of XSS attacks.
2. Cross site request forgery (CSRF) protection: CSRF attacks allow a malicious user to execute the credentials of another user without user's consent. Therefore, Django provides security against CSRF attacks using built-in CSRF protection, provided the developer has enabled and used it wherever appropriate.
3. SQL injection protection: SQL injection attack is attack where the malicious user can execute the arbitrary SQL query on the shared database. Django's query sets are protected from SQL injection by providing separation of query's SQL code and parameters.
4. Clickjacking protection: It is a type of attack any malicious website can be rendered on the top of action the users think they are performing.

d. Technology Used

SWADESH components are implemented using python-based Django framework, which is widely used in the development of various famous sites.

III. Overview of the functionalities

SWADESH provides the user a unified platform to search and access multimodal neuroimaging data of healthy and diseased categories along with visualization and analytical tools as depicted in Figure 1. These functionalities are explained in the following sections.

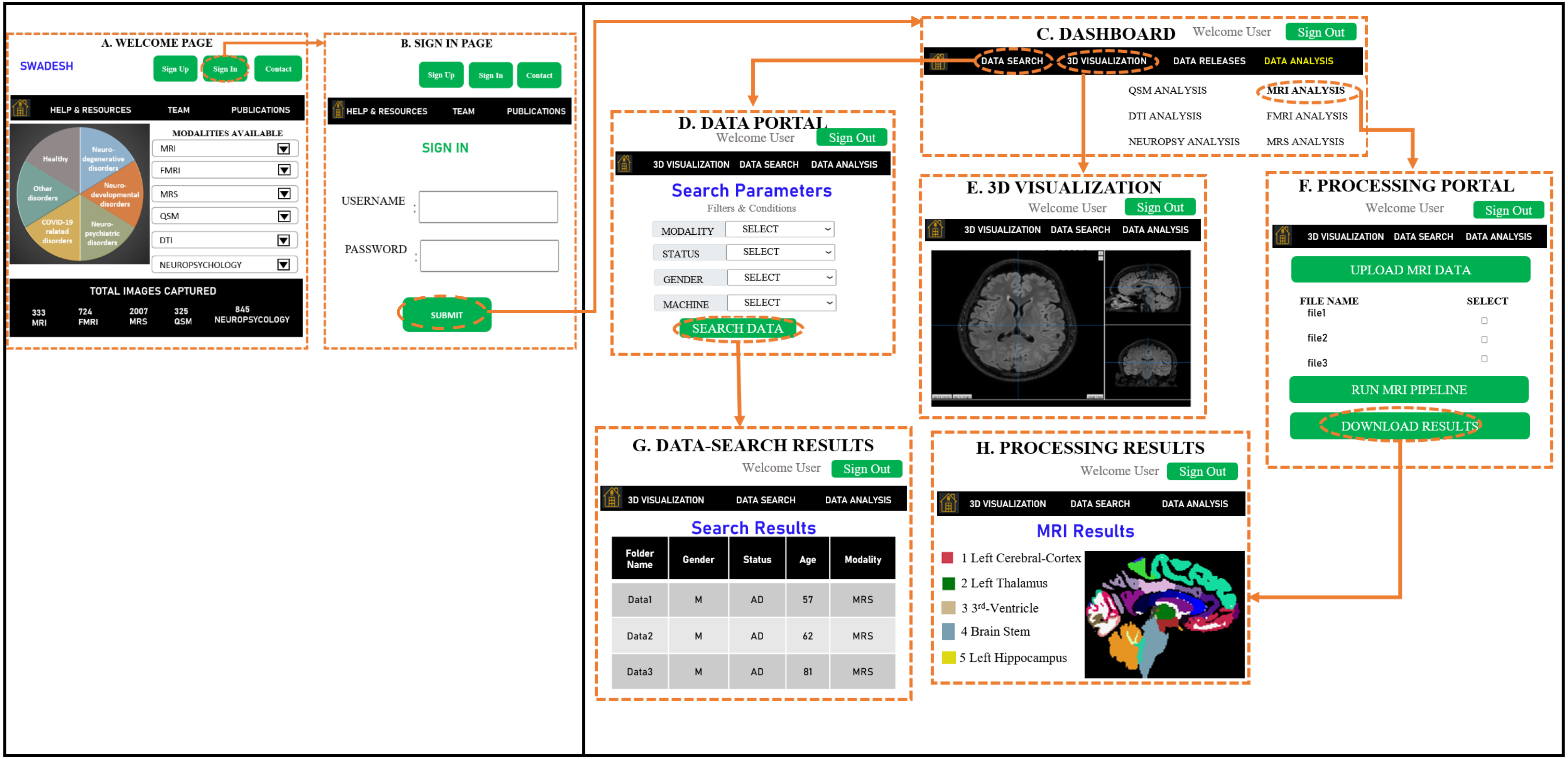

Figure 1: Web-based SWADESH system, consists of various stages (A) Front-page provides the introduction of SWADESH webpage, (B) In the second stage, the user can sign in for accessibility of SWADESH, (C) After logging in, the third page provides the dashboard to the user along with options of data search, visualize and analyze multimodal neuroimaging data, (D) A sub-page of the third page which provides the user option to search data corresponding to various modalities, status, gender and machine (E) image viewer to display the 3-dimensional picture of the image to perform a visual inspection of image quality (F) Processing portal for running modality-specific pipeline for the data to be analyzed (For demonstration here, MRI pipeline is selected), (G) The web-page of data search results as per specified parameters in (D), (H) Processing results obtained after running the modality-specific analytic pipeline on uploaded data in (F).

2. Getting started

SWADESH platform can be accessed at: http://swadesh.nbrc.res.in/. This platform provides multimodal neuroimaging data along with analytical tools for processing. The platform supports data of: MRI, MRS, fMRI, QSM, DTI and neuropsychological test scores. Figure 2 provides the user-flow of the SWADESH web-based platform.

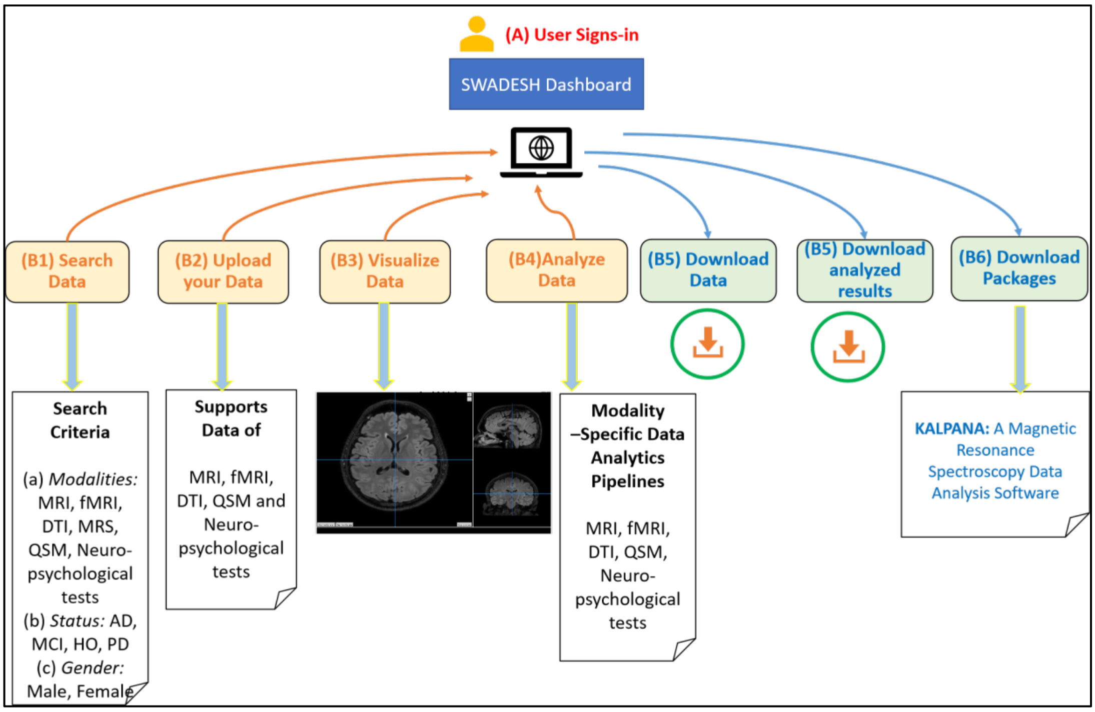

Figure 2: Overview of SWADESH platform. The user first needs to sign-in for accessibility of SWADESH After signing in, the platform provides the user with options of (B1) search data, (B2) upload you data, (B3) visualize data, (B4) analyze data, (B5) download data, (B6) download analyzed results and (B7) download additional packages like KALPANA for MRS processing.

An account can be created to access the platform by using "Sign-Up" button. User can sign-in using "Sign-In" button by filling in assigned username and password as depicted in Figure 3.

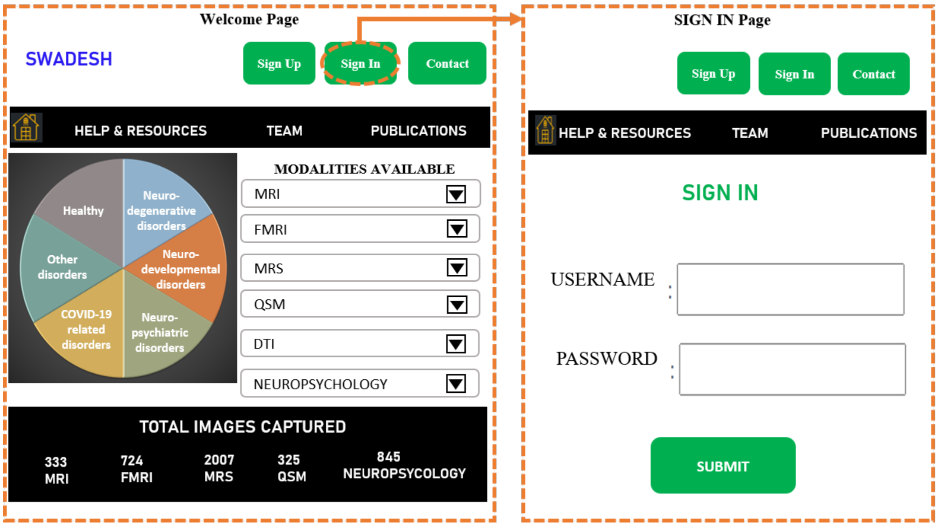

Figure 3: Welcome and sign-in page of SWADESH

When a user sign-in with valid credentials, a dashboard page (Figure 4) is opened. This page represents the status of the database in terms of data count and status. The user can avail the options of data access, visualization and analytics as per the requirement.

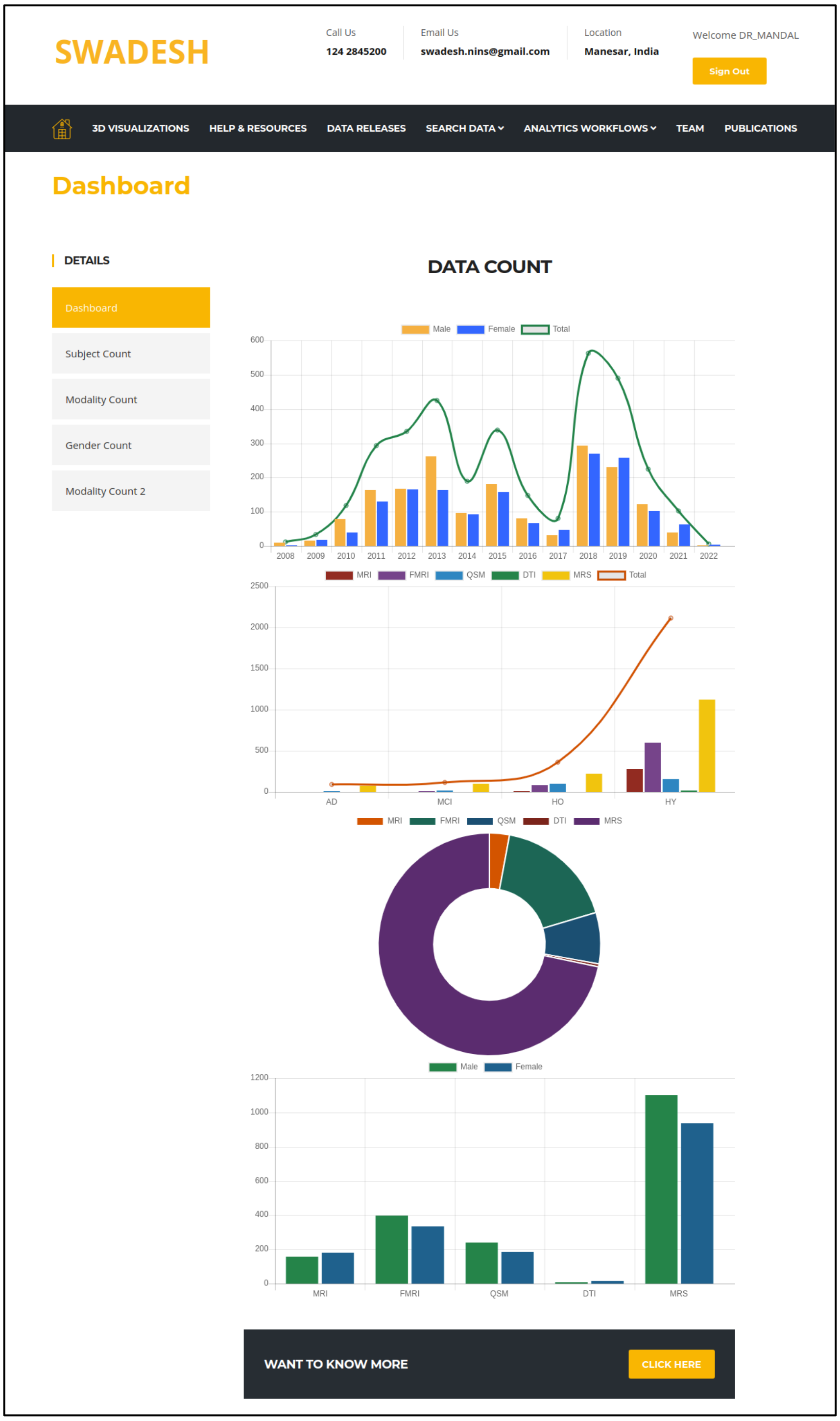

Figure 4: Dashboard page after signing-in

3. Data Search

Using "Search Data" option, user can access the multimodal neuroimaging and behavioral data for various brain disorders. The user needs to click on "Advanced Search" option under "Search Data" to specify the filters and conditions of the required data. This is depicted in Figure 5. The specifications' options are: type of modality, gender, status, machine and country.

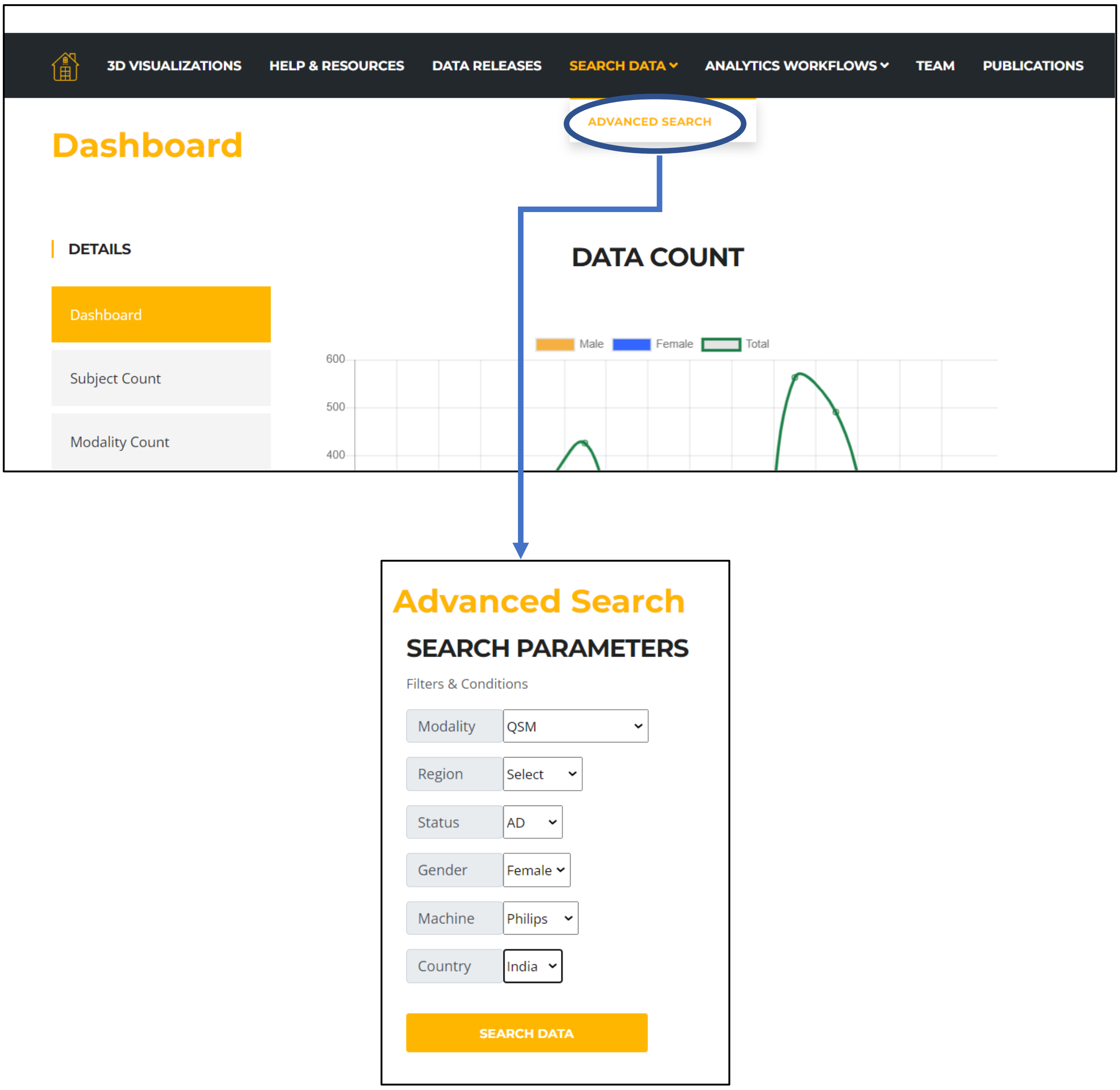

Figure 5: Data Search option and advanced search dialog for specifying the search parameters

After specifying the parameters, click on the "search data" button below to access the data. The user can then download the specified data by clicking on "Download Raw Data" (Figure 6). The data is downloaded in zip format and appears as shown in Figure 7.

RESULTS

Sr.No.	ID_Code	Gender	Status	Age_during_scan_time	Modality	Region	Machine	Country
1	111zhgb	F	AD	66	QSM		Philips	India
2	111edaf	F	AD	72	QSM		Philips	India
3	111ezeh	F	AD	65	QSM		Philips	India
4	111ezgz	F	AD	81	QSM		Philips	India
5	111ezge	F	AD	75	QSM		Philips	India
6	111ezhg	F	AD	82	QSM		Philips	India
7	111ezhc	F	AD	68	QSM		Philips	India
8	111eedf	F	AD	79	QSM		Philips	India
9	111eedg	F	AD	76	QSM		Philips	India
10	111eeih	F	AD	74	QSM		Philips	India

DOWNLOAD RAW DATA

Figure 6: Advanced search results as per the specifications provided by the user (as in Figure 5)

Location: /copyloc_DR_MANDAL/

Name	Size	Type	Modified
111zhgb	396.1 MB	Folder	19 October 2022, 06:05
111ezhg	396.1 MB	Folder	19 October 2022, 06:05
111ezhc	396.1 MB	Folder	19 October 2022, 06:05
111ezgz	396.1 MB	Folder	19 October 2022, 06:05
111ezge	396.1 MB	Folder	19 October 2022, 06:05
111ezeh	396.1 MB	Folder	19 October 2022, 06:05
111eeih	396.1 MB	Folder	19 October 2022, 06:06
111eedg	396.1 MB	Folder	19 October 2022, 06:05
111eedf	398.8 MB	Folder	19 October 2022, 06:05
111edaf	396.1 MB	Folder	19 October 2022, 06:05

Figure 7: Downloaded data obtained as per the specifications given by the user (as in Figure 5)

4. 3D Visualizations

The 3-dimensional image viewer is also provided in the SWADESH platform for visual inspection of the data in axial, sagittal and coronal view. Using "3D Visualizations" option on the dashboard, the user can view an MRI image by selecting the "file option" and specifying the file as shown in Figure 8. Once the image is selected, the viewer shows the axial, sagittal and coronal views (see Figure 9).

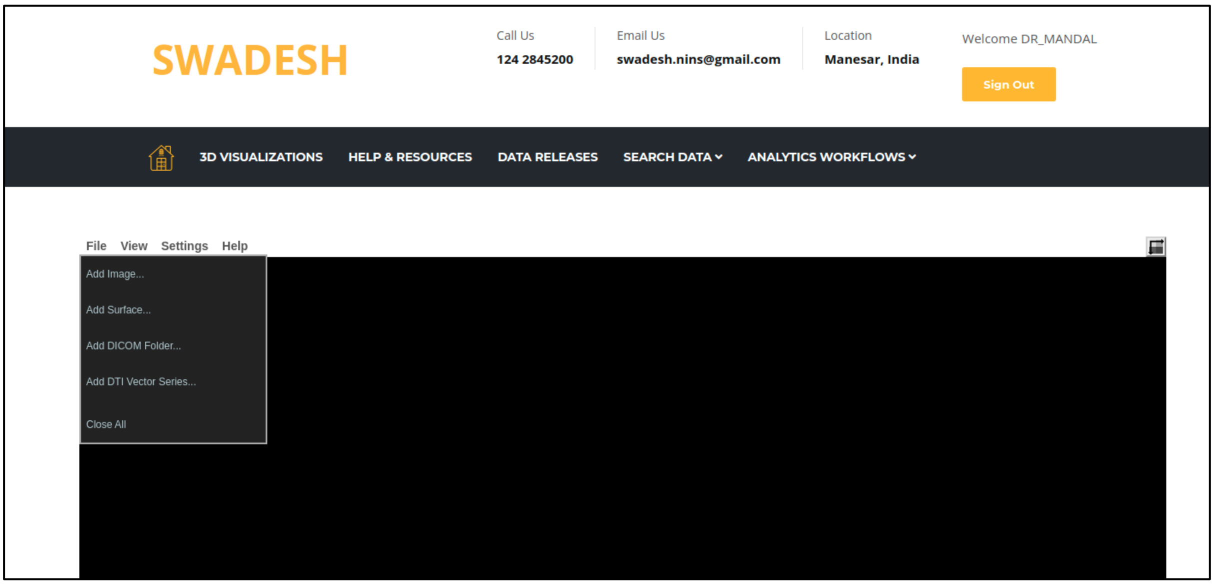

Figure 8: 3D Visualization option and its interface

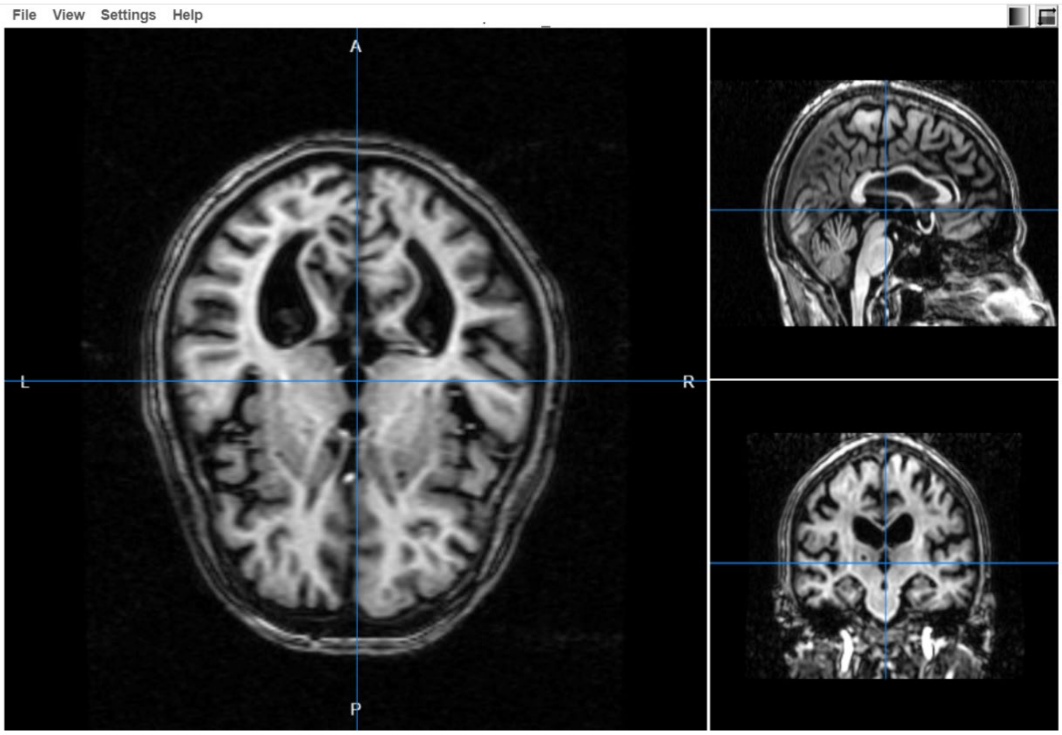

Figure 9: 3-dimensional view of the selected file in the image viewer

5. Analytics Workflows

In SWADESH, the data analytical tools are available to process MRI, fMRI, DTI, QSM and Neuropsychological scores. The user can use the option of "Analytics Workflows" on the dashboard to process various types of neuroimaging data. By clicking on the "Analytics Workflows", user can select the analytical pipelines for various modalities (Figure 10).

For demonstration, we are showing the use of MRI analytical pipeline. From the drop-down list under "Analytics Workflows", user can select MRI Analysis (Figure 10).

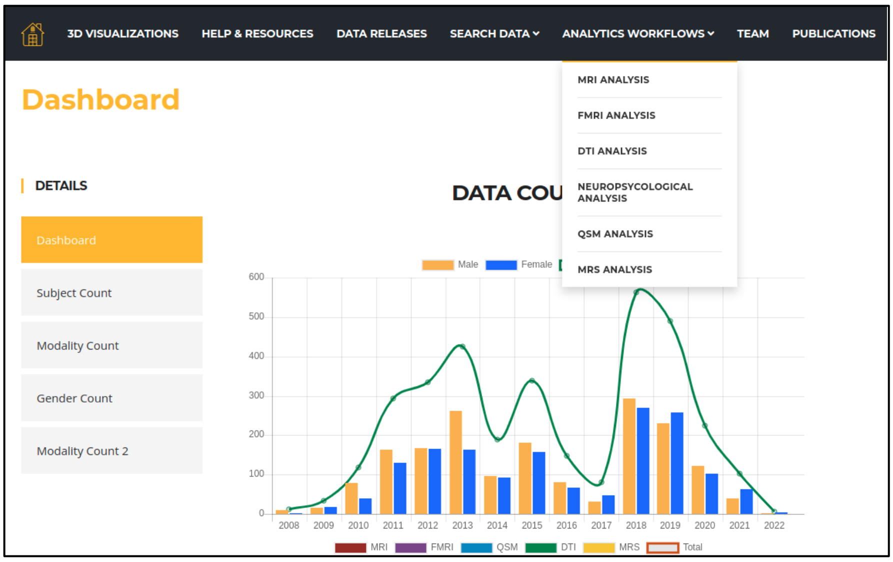

Figure 10: Analytics Workflows options on the dashboard to process modality-wise data

When the user clicks on "MRI analysis", a window as shown in Figure 11 (a) opens up, where the user can upload the MRI data to be processed and download the results. On clicking "Upload MRI files" (Figure 11 (a) , user can name the collection (say "MRI_test"), select and upload the MRI files for processing.

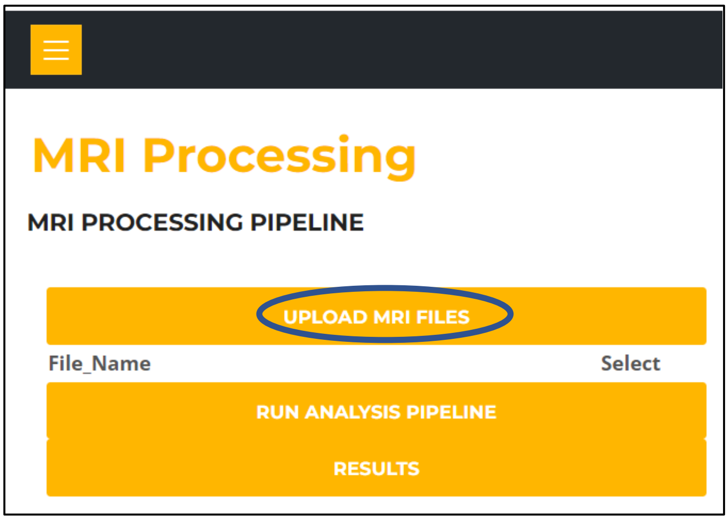

(a)

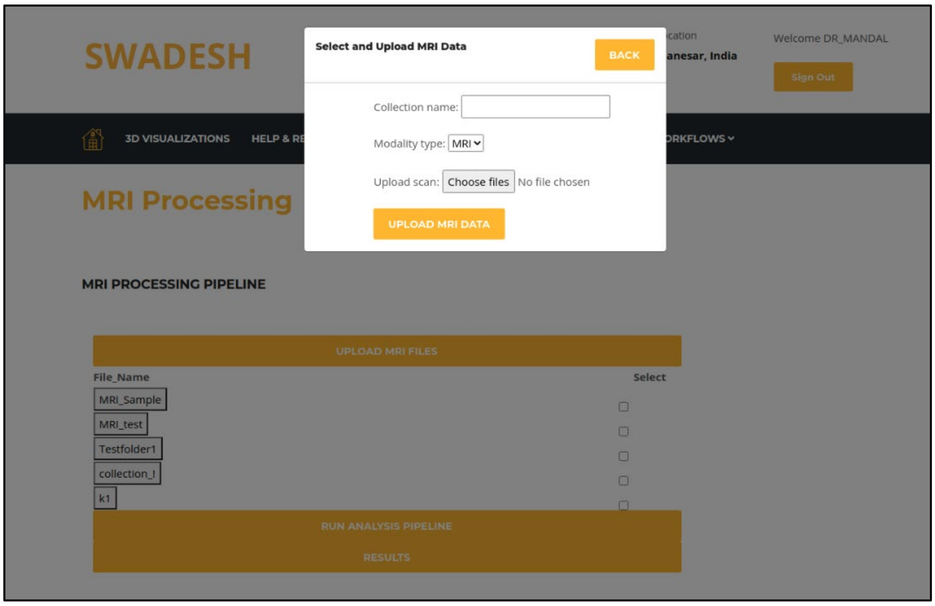

(b)

Figure 11: (a) MRI Analysis dialog on selecting MRS analysis under "Analytics Workflows"b) Pop-up dialog after clicking "Upload MRI files"

Once the data gets uploaded, user need to select (tick) the collection name for running the MRI analytics pipeline on the selected collection. Then, the user can click on "Run analysis pipeline", to run the processing as shown in Figure 12.

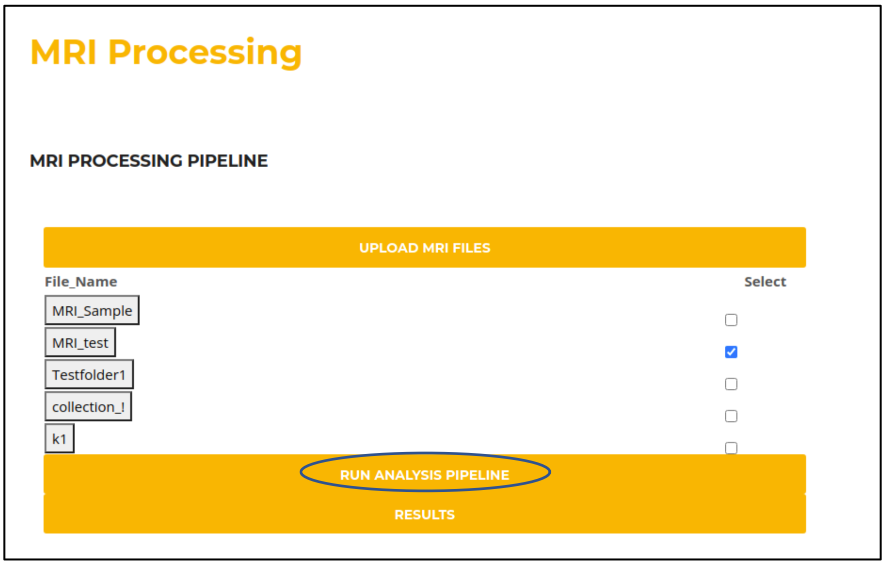

Figure 12: MRI Analysis window for processsing MRI data

Once the processing is complete, user can click on "Results" button to download the results (Figure 13).

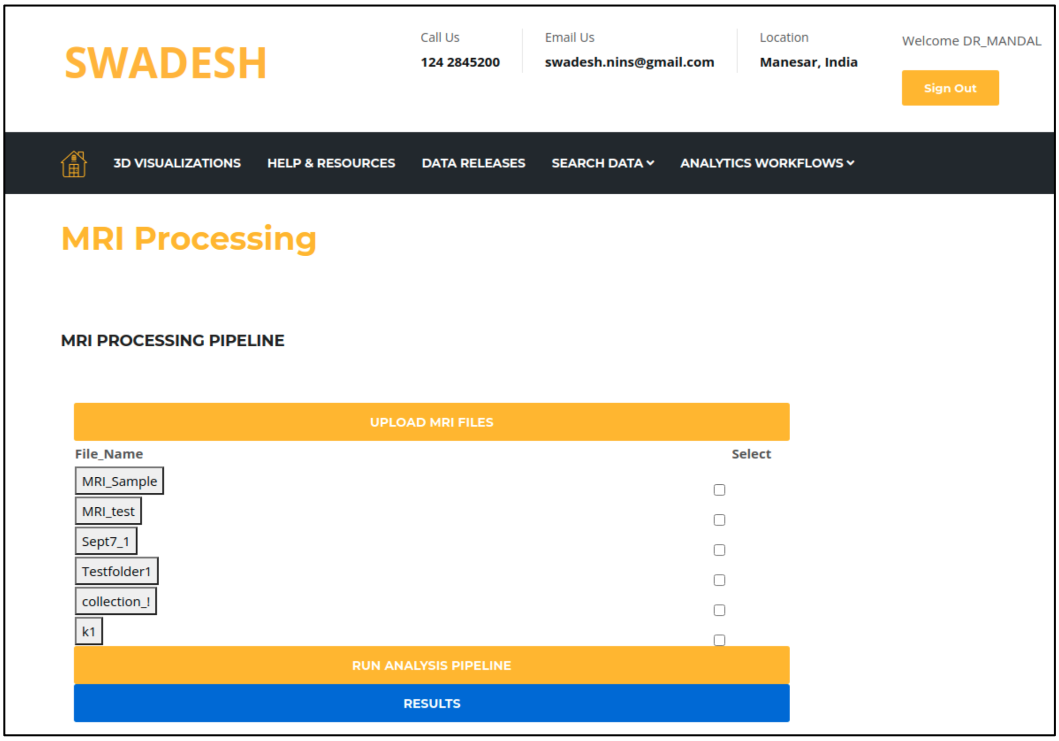

Figure 13: MRI Analysis window for downloading results

On clicking the results, a zip folder is downloaded that contains all the processed files. For a particular collection, the processed files of MRI contain the label generated files as depicted in Figure 14.

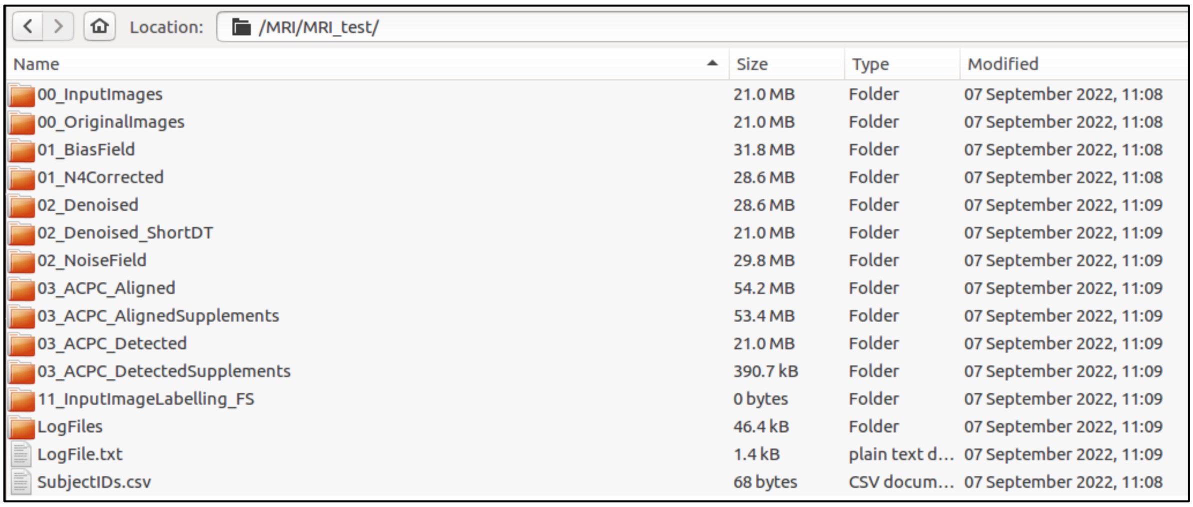

Figure 14: Processed files obtained after running MRI processing pipeline

In a similar way, users can upload the data and process it for other neuroimaging analytic pipelines. Following figures show the processed files obtained after running these analytical workflows.

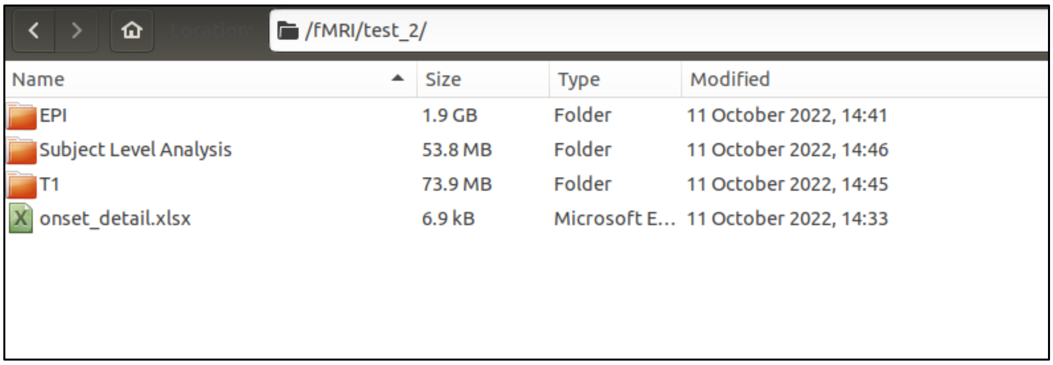

Figure 15: Processed files obtained after running fMRI processing pipeline

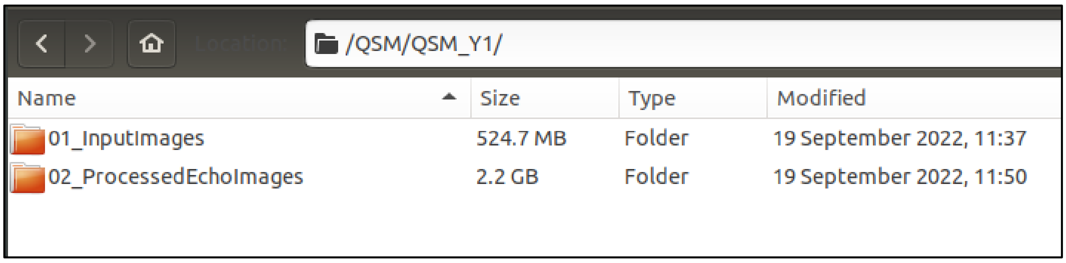

Figure 16: Processed files obtained after running QSM processing pipeline

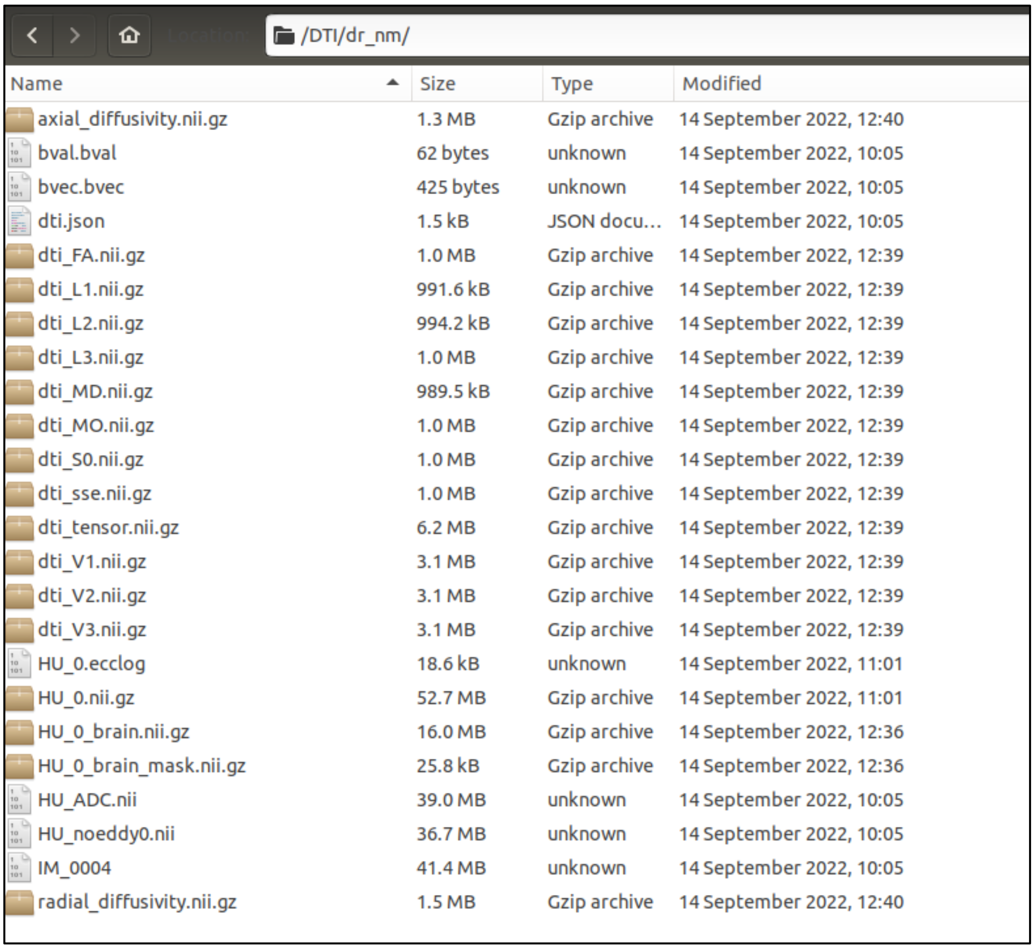

Figure 17: Processed files obtained after running DTI processing pipeline

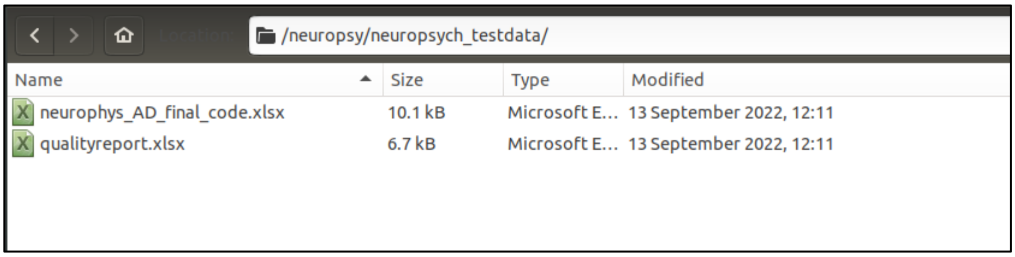

Figure 18: Processed files obtained after running Neuropsychological analysis pipeline

6. Additional Features

In SWADESH, the link to various GUI-based data analytical tools developed by the NINS Lab are also provided as additional resources. Currently, The package includes: KALPANA for MRS processing.

7. Help and technical support

If you have any questions about using SWADESH or comments on this guide, you can email support at: swadesh.nins@gmail.com

8. Citation

If you use SWADESH to process or access data, please cite the following publications:

Mandal PK, Jindal K, Roy S, Arora Y, Sharma S, Joon S, Goel A, Ahasan Z, Maroon JC, Singh K, Sandal K, Tripathi M, Sharma P, Samkaria A, Gaur S and Shandilya S (2023) SWADESH: a multimodal multi-disease brain imaging and neuropsychological database and data analytics platform. Front. Neurol. 14:1258116.doi: 10.3389/fneur.2023.1258116

Mandal PK, Jindal K, Maroon JC, Chhikara R, Samkaria A, Joshi M, Roy S, Arora Y. (2023). Brain Imaging Databases. ACS Chemical Neuroscience.

Mandal, P. K., & Perry, G. (2022). SWADESH: A Comprehensive Platform for Multimodal Data and Analytics for Advanced Research in Alzheimer's Disease and Other Brain Disorders. *Journal of Alzheimer's Disease*, *85*(1), 1-5. doi: 10.3233/JAD-215354

9. Data Specifications

The following table summarizes the data details available on the SWADESH portal:

Sr. No.	STATUS	MRI	MRS	QSM	fMRI	Neuropsych Data
**1.**	HY	474<br>(475)	339<br>(1004)	92<br>(92)	108<br>(122)	211<br>(211)
**2.**	HO	78<br>(80)	159<br>(284)	57<br>(57)	28<br>(29)	190<br>(191)
**3.**	MCI	17<br>(18)	58<br>(129)	15<br>(15)	08<br>(08)	17<br>(17)
**4.**	AD	37<br>(38)	100<br>(207)	22<br>(22)	17<br>(17)	39<br>(39)
**5.**	PD	35<br>(35)	32<br>(78)	27<br>(27)	0	41<br>(41)

(The numbers on top of each cell represent the head count and numbers in parenthesis represents the data records)

* Head count represents the total number of participants, while data records encompass data collected from various modalities, regardless of whether it pertains to the same individual. For instance, MRS can be acquired from multiple regions like hippocampus, frontal cortex, parietal cortex etc and for different metabolites like Glutathione, GABA, and others. In this case, there will be multiple data records for one person (single head count).

Scanner: Philips Achieva 3T MRI scanner

The following table provides a brief description of the data specifications

S. No.	Modality	Data Format	Description
1.	MRI	.par, .nifti	T1, T2-weighted, and Flair MRI images
2.	fMRI	.par, .nifti	T2* Image<br>EPI (Echo Planar Imaging) Sequence
3.	DTI	dicom	DWISE Pulse sequence with Diffusion acquisition contrast
4.	QSM	dicom	Gradient echo scans with multi fast field echo (mFFE) sequence
5.	MRS	.spar	Glutathione & GABA data acquired on different brain regions using MEGA-PRESS sequence
6.	Neuropsychological data	Excel Sheet	Test scores list
